# Phase II Clinical Study of Adebrelimab and Bevacizumab Combined With Cisplatin/Carboplatin in Patients With Triple-Negative Breast Cancer With Brain Metastases (ABC Study)

**DOI:** 10.1200/JCO-25-02021

**Published:** 2026-03-04

**Authors:** Ting Li, Teng Zhou, Biyun Wang, Zhonghua Tao, Mingchuan Zhao, Leiping Wang, Juan Jin, Yannan Zhao, Chengcheng Gong, Jun Cao, Haotao Miao, Wenxia Peng, Jianfei Wang, Min Jin, Xichun Hu, Jian Zhang

**Affiliations:** ^1^ Department of Medical Oncology, Fudan University Shanghai Cancer Center, Shanghai, China; ^2^ Department of Oncology, Shanghai Medical College, Fudan University, Shanghai, China; ^3^ Phase I Clinical Trial Center, Fudan University Shanghai Cancer Center, Shanghai, China; ^4^ Jiangsu Hengrui Pharmaceuticals Co.,Ltd, Shanghai, China

## Abstract

**PURPOSE:**

Brain metastases (BMs) of triple-negative breast cancer (TNBC) are lethal, often associated with a limited life span and lack of effective antitumor agents. Here we reported a triple combination therapy consisting of adebrelimab, bevacizumab, and cisplatin/carboplatin in BMs of TNBC.

**METHODS:**

This phase II clinical trial involved patients with TNBC with active BMs. Participants were administered with adebrelimab, bevacizumab, and cisplatin/carboplatin until disease progression or unacceptable toxic effects. The primary end point was the objective response rate in CNS (CNS-ORR) according to the Response Assessment in Neuro-Oncology BMs criteria, and the secondary end points included the clinical benefit rate in CNS (CNS-CBR), progression-free survival (PFS), overall survival (OS), the first progression site, and safety.

**RESULTS:**

A total of 35 patients were enrolled and treated, and the median lines of previous treatment were 2 (range, 0-4). The confirmed CNS-ORR was 77.1% (27/35, 95% CI, 59.9 to 89.6), and the CNS-CBR was 80.0% (28/35, 95% CI, 63.1 to 91.6). The median overall PFS was 8.3 months (95% CI, 5.8 to 11.5), whereas the median CNS-PFS was 10.3 months (95% CI, 7.4 to 14.3) and the median OS was 21.1 months (95% CI, 13.2 to not reached). Among the 28 patients who progressed, progression was intracranial-only in 32.1% (9/28) patients, extracranial-only in 35.7% (10/28) patients, and both in 32.1% (9/28) patients. The incidence of grade ≥3 treatment-related adverse events was 65.7% (23/35). Treatment-related serious adverse events occurred in five patients (14.3%), and no treatment-related deaths were reported.

**CONCLUSION:**

The combination of adebrelimab, bevacizumab, and cisplatin/carboplatin was the first regimen to demonstrate promising intracranial antitumor activity and prolonged PFS and CNS-PFS, along with a manageable safety profile, warranting further investigation.

## INTRODUCTION

Breast cancer remains the most common malignancy among women worldwide, with triple-negative breast cancer (TNBC) known for its aggressive nature and high risk of spreading to the brain.^1^ Approximately one third of patients with TNBC develop brain metastases (BMs), with a median overall survival (OS) of 6-12 months, shorter than that of patients with advanced breast cancer without BMs.^2-4^ Although radiotherapy and surgical resection are the primary treatment options for TNBC BMs, local recurrences typically occur at a median time of approximately 5 months after treatment.^5^

**CONTEXT**

**Key Objective**
To evaluate the efficacy and safety of adebrelimab, bevacizumab, and platinum chemotherapy in patients with triple-negative breast cancer (TNBC) and active brain metastases (BMs).
**Knowledge Generated**
The triplet treatment of adebrelimab, bevacizumab, and cisplatin/carboplatin demonstrated a promising intracranial antitumor activity as well as prolonged intracranial survival benefits in patients with TNBC and BMs. In addition, the safety profile was manageable.
**Relevance *(K.D. Miller)***
CNS involvement is a common and feared complication of metastatic TNBC. Despite the encouraging initial results, randomized trials are needed before this regimen can be widely recommended.*
*Relevance section written by *JCO* Associate Editor Kathy D. Miller, MD, FASCO.

For TNBC BMs, systemic treatment options are limited and primarily follow the same strategies as for metastatic TNBC, which yield unsatisfactory outcomes due to challenges with drug delivery across the blood-brain barrier.^6-8^ Chemotherapy combined with immune checkpoint inhibitors is the standard first-line therapy for PD-L1–positive metastatic TNBC; however, clinical trials have enrolled few patients with BMs or only patients with stable BMs, leading to a lack of understanding of immunotherapies' contribution in this subgroup.^9,10^ The advent of antibody-drug conjugates (ADCs) has brought revolutionary advances in the treatment of metastatic TNBC, including human epidermal growth factor receptor 2 (HER2) ADCs (eg, trastuzumab deruxtecan [T-DXd] for HER2-low breast cancer) and trophoblast cell surface antigen 2 ADCs (eg, sacituzumab govitecan [SG] and sacituzumab tiragolomab).^11-13^ Although ADCs are being investigated for breast cancer BMs, most existing evidence pertains to HER2-positive subtypes, which have demonstrated significant intracranial activity.^14,15^ However, evidence remains insufficient regarding their effectiveness against BMs in TNBC. Current evidence suggests that platinum-based chemotherapy can elicit intracranial responses in patients with BMs, and the addition of bevacizumab may enhance intracranial response rates and further improve progression-free survival (PFS).^16-19^ Notably, emerging data from non–small cell lung cancer with BMs have shown that the triple combination of immunotherapy, platinum chemotherapy, and bevacizumab confers a prolonged PFS.^20^ These findings underscore the potential of combining immunotherapy with platinum chemotherapy and bevacizumab for TNBC BMs and highlight the need for further investigations.

Adebrelimab is a high-affinity, humanized IgG4 monoclonal antibody targeting PD-L1. In the CAPSTONE-1 study, adebrelimab combined with carboplatin and etoposide significantly improved OS in patients with extensive-stage small cell lung cancer (ES-SCLC).^21^ Another study of adebrelimab plus chemotherapy followed by thoracic radiotherapy—including one third of patients with BMs—reported the longest OS to date.^22^ Furthermore, real-world data have further shown that adebrelimab-based first-line treatment exhibits clinical activity in patients with ES-SCLC with BMs.^23^

Therefore, we conducted the ABC study (also called the BCBM-001 study), which is the first prospective study to investigate the activity and safety of adebrelimab and bevacizumab combined with platinum chemotherapy (cisplatin/carboplatin) in TNBC with BMs.

## METHODS

### Study Design and Participants

The ABC study was an investigator-initiated, single-arm, phase II clinical trial conducted at Fudan University Shanghai Cancer Center in China. Eligible participants were age 18-70 years, with an Eastern Cooperative Oncology Group performance status of 0-2. All patients had pathologically confirmed hazard ratio-negative and HER2-negative breast cancer, with evidence of local recurrence or metastasis, and were deemed unsuitable for curative surgery or radiation therapy to the primary breast tumor and regional lymph nodes. Patients with untreated or progressive BMs and at least one measurable intracranial lesion with a longest diameter of 1.0 cm or greater per Neuro-Oncology BMs criteria (Response Assessment in Neuro-Oncology BMs [RANO-BM]) were eligible. Patients were excluded if they had previous exposure to bevacizumab or anti–PD-1/PD-L1 therapies. Previous platinum therapy was allowed only for platinum-sensitive disease (no progression within four cycles of platinum and a treatment-free interval more than 3 months before progression). More details are provided in the study protocol (Appendix, online only).

The trial was designed and conducted in accordance with the Declaration of Helsinki and the Guidelines for Good Clinical Practice, and received approval from the Ethics Committee of Fudan University Shanghai Cancer Center. All participants provided written informed consent. This trial is registered at clinical trials.gov (identifier: NCT04303988).

### Procedures

Eligible patients received intravenous administration of the following drugs: adebrelimab (20 mg/kg, D1, once every 3 weeks), bevacizumab (7.5 mg/kg, D1, once every 3 weeks), and cisplatin (75 mg/m^2^, D1, once every 3 weeks) or carboplatin (AUC = 5, D1, once every 3 weeks). The choice of cisplatin or carboplatin was determined by previous exposure, with the alternative selected if one was used before; if both or neither were used, selection was at the investigator's discretion. Treatment was continued until disease progression, unacceptable toxicity, withdrawal of informed consent, or discontinuation of the study as determined by investigators, whichever occurred first.

Tumor response assessments were conducted at baseline and every two cycles using enhanced magnetic resonance imaging (MRI) or computed tomography. Imaging evaluation of intracranial lesions was based on the RANO-BM criteria, whereas extracranial lesions were assessed according to the RECIST guidelines version 1.1 (RECIST 1.1). The first intracranial/extracranial partial response (PR) or complete response (CR) had to be confirmed at least 4 weeks later. OS was followed up every 3 months until death, loss to follow-up, or completion of the study. Safety assessments were conducted at screening and each study visit, and adverse events (AEs) were graded according to the National Cancer Institute's Common Terminology Criteria for Adverse Events, version 5.0.

### Outcomes

The primary end point was objective response rate in the CNS (CNS-ORR), defined as the proportion of patients with the best intracranial response of CR or PR. The secondary end points included clinical benefit rate in the CNS (CNS-CBR), defined as the percentage of patients who experience intracranial CR, PR, or stable disease (SD) ≥24 weeks, PFS (defined as the time from the first dose of treatment to death or disease progression), OS (defined as the time from the first dose of treatment to death for any cause), site of first progression (defined as the first lesion to progress), and safety.

### Exploratory Analyses

We assessed PD-L1 expression, androgen receptor (AR), CD8, and forkhead box C1 protein (FOXC1) in tumor samples by immunohistochemistry (IHC). The expression of PD-L1 was performed using the PD-L1 IHC 22C3 pharmDx assay (Agilent Technologies, Carpinteria, CA). The tumors were divided into four molecular subtypes according to the Fudan TNBC classification system: immunomodulatory (IM), basal-like immune-suppressed, luminal AR, and mesenchymal, based on the expression of AR, CD8, and FOXC1.^24,25^

### Statistical Analyses

The sample size was calculated based on the primary end point of CNS-ORR using a Simon's^26^ optimal two-stage design. Given the limited data reported at the time of ABC study design, referring to the historical data on platinum-based regimen in solid tumor BMs and iniparib-based regimen in TNBC BMs, the null hypothesis for CNS-ORR was set at 14%,^27,28^ and the alternative hypothesis for CNS-ORR was set at 34%, with a one-sided α of .05 and a power of 80%. Accounting for a 10% dropout rate, a total of 35 subjects were required to be enrolled.

Efficacy assessments were conducted in full analysis set (patients who received at least one cycle of study treatment). Safety analyses were conducted in safety set (patients who received at least one cycle of study treatment and had available safety data).

Continuous data were presented as median (range), whereas categorical data were summarized as frequency (percentage). The 95% CIs of ORR and CBR were estimated using the Clopper-Pearson method. PFS and OS were calculated using the Kaplan-Meier method, and their 95% CIs were estimated using the Brookmeyer-Crowley method. Statistical analyses were performed using SPSS software (version 27.0).

## RESULTS

### Baseline Characteristics

**FIG 1.**
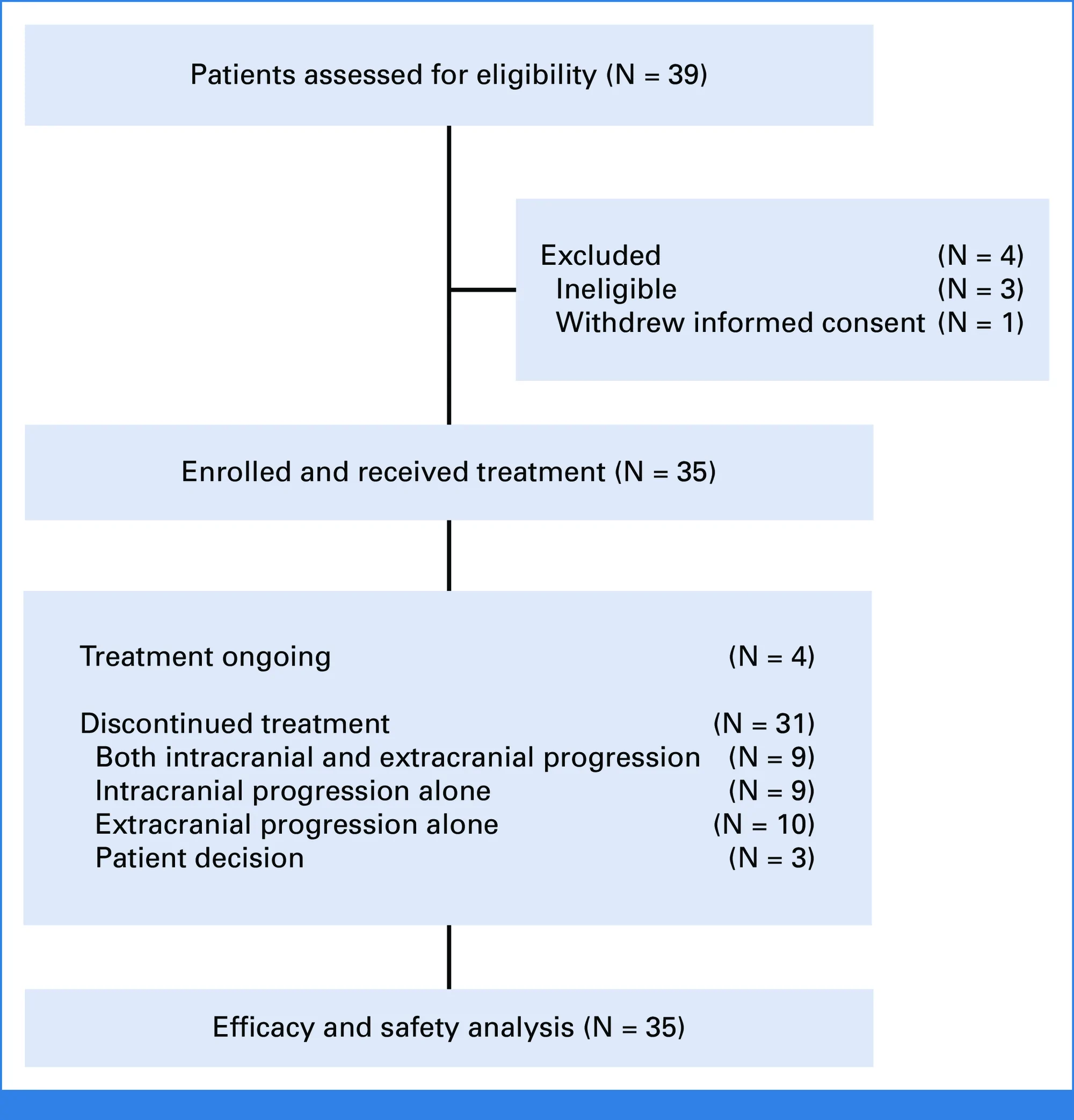
The ABC trial flow diagram. Three patients withdrew from the study for the following reasons: one due to restrictions during the COVID-19 pandemic, one due to travel burden, and one opted for CNS radiotherapy despite no evidence of intracranial progression.

Between July 3, 2020, and October 18, 2024, a total of 39 patients were screened for eligibility, and 35 patients were enrolled to receive the treatment of adebrelimab, bevacizumab, and a platinum agent (30 [85.7%] received cisplatin and five [14.3%] received carboplatin). As of the data cutoff on June 10, 2025, treatment was ongoing in four patients (11.4%), whereas 28 patients (80.0%) discontinued treatment due to disease progression and three patients (8.6%) due to patient decision (Fig 1). All enrolled patients were female, with a median age of 50 years (range, 36-64 years). Among the enrolled patients, 14.3% (5/35) had received previous CNS-directed radiation and 42.9% (15/35) had neurologic symptoms at baseline. The median previous lines of therapy for metastatic disease were 2 (range, 0-4). Patients' characteristics at baseline are shown in Table 1.

**TABLE 1.** Baseline Characteristics

Characteristics	Patients (N = 35)
Age, years, median (range)	50 (36-64)
ECOG PS, No. (%)	
1	34 (97.1)
2	1 (2.9)
PD-L1 status, No. (%)	
CPS <1	13 (37.1)
CPS ≥1	11 (31.4)
Unknown	11 (31.4)
Fudan TNBC classification, No. (%)	
IM	8 (22.9)
Non-IM	22 (62.9)
BLIS	14 (40.0)
LAR	4 (11.4)
MES	4 (11.4)
Unknown	5 (14.3)
Presence of neurologic symptoms at baseline, No. (%)	
Yes	15 (42.9)
No	20 (57.1)
No. of BMs, No. (%)	
1-3	19 (54.3)
4-9	12 (34.3)
≥10	4 (11.4)
Metastatic disease from diagnosis of breast cancer, months, median (range)	24.0 (0.1-139)
BMFS, months, median (range)	
BMFS from diagnosis of breast cancer	32.2 (0.1-227.3)
BMFS from diagnosis of metastatic disease	1.5 (0-88.2)
Status of disease, No. (%)	
CNS disease only	5 (14.3)
Both CNS and extracranial disease	30 (85.7)
Visceral metastases (excluding BMs), No. (%)	
Yes	20 (57.1)
No	15 (42.9)
Sites of disease (excluding BMs), No. (%)	
Lung	18 (51.4)
Liver	4 (11.4)
Bone	17 (48.6)
Lymph node	20 (57.1)
Status of BMs, No. (%)	
Untreated	28 (80.0)
Previous local therapy for BMs	7 (20.0)
Surgery only	2 (5.7)
WBRT/SRT/SRS only	2 (5.7)
Surgery + WBRT/SRT/SRS	3 (8.6)
Time from previous CNS-directed radiation to enrollment, months, median (range)	10.0 (3.7-24.8)
No. of previous systemic therapy lines in metastatic setting, No. (%)^a^	
0	9 (25.7)
1	8 (22.9)
2	12 (34.3)
≥3	6 (17.1)
Previous lines of therapy for metastatic breast cancer, median (range)	2 (0-4)
Previous systemic therapy for metastatic breast cancer, No. (%)	
Chemotherapy	33 (94.3)
Endocrine therapy	6 (17.1)
mTOR inhibitors	3 (8.6)
CDK4/6 inhibitors	2 (5.7)
PARP inhibitors	1 (2.9)
Antibody-drug conjugate	1 (2.9)
Previous platinum chemotherapy, No. (%)	
Yes	14 (40.0)
No	21 (60.0)

NOTE. Fudan TNBC classification: IM, LAR, BLIS, MES.

Abbreviations: BLIS, basal-like immune-suppressed; BMFS, brain metastasis–free survival; BMs, brain metastases; CDK4/6 inhibitors, cyclin-dependent kinase 4/6 inhibitor; CPS, combined positive score; ECOG PS, Eastern Cooperative Oncology Group performance status; IM, immunomodulatory; LAR, luminal androgen receptor; MES, mesenchymal; mTOR inhibitors, mammalian Target of Rapamycin inhibitors; PARP inhibitors, Poly(ADP-ribose) polymerase inhibitors; SRS, stereotactic radiosurgery; SRT, stereotactic radiotherapy; TNBC, triple-negative breast cancer; WBRT, whole brain radiation therapy.

^**a**^ Recurrence occurring within 12 months of completing (neo)adjuvant therapy was considered as one line of therapy.

### Efficacy

**FIG 2.**
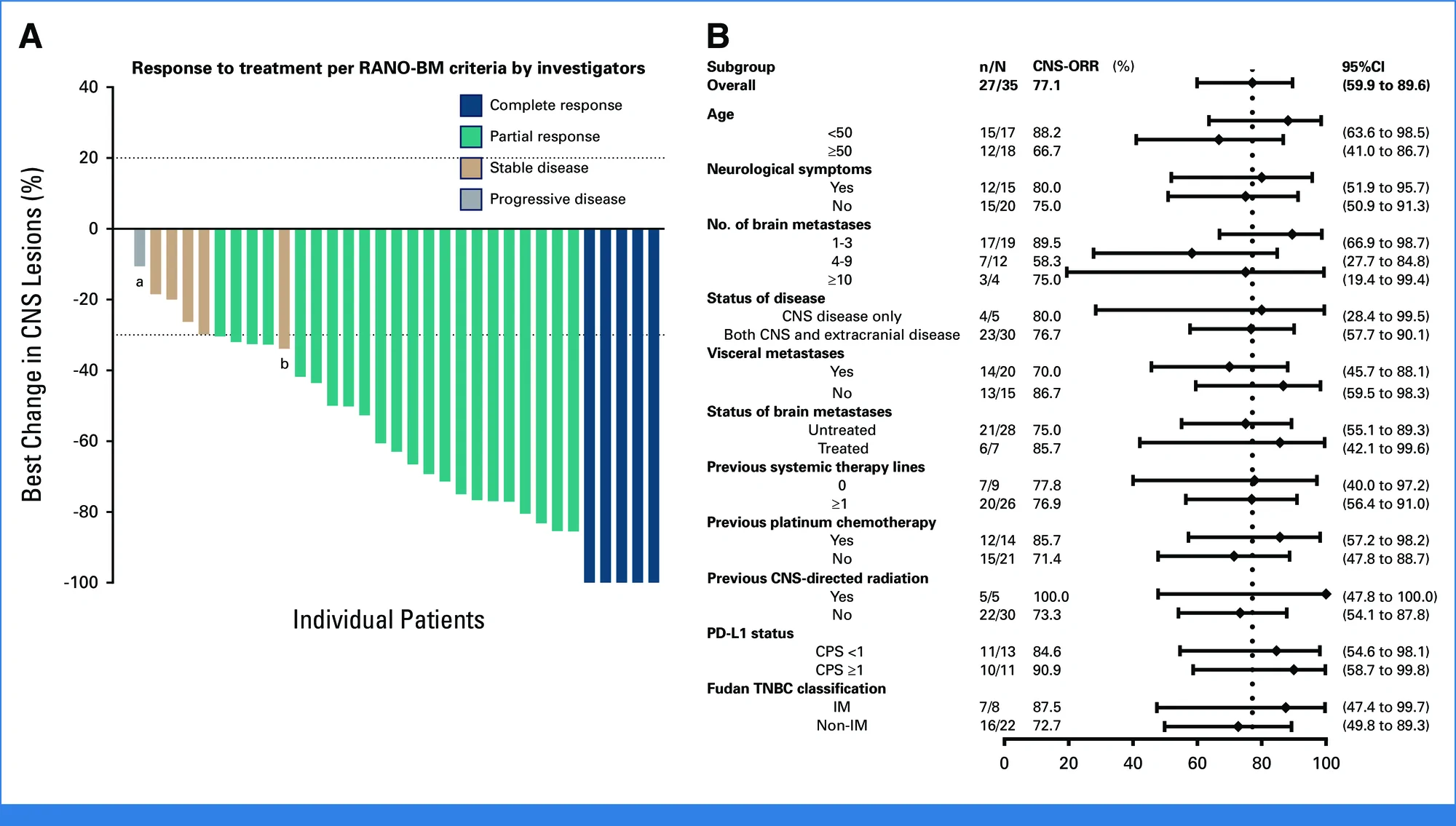
Waterfall plot for best change in brain target lesions from baseline and post hoc subgroup analyses of CNS-ORR. (A) Waterfall plot for best change in brain target lesions from baseline according to RANO-BM criteria. The dashed lines at 20% and –30% denote the thresholds for progressive disease and PR, respectively. One patient (patient 028) was excluded because she received only one cycle of treatment, with progression of clinical symptoms, and was assessed as progressive disease. ^a^Patient 010, about 10.6% regression of the intracranial lesion was observed, but new lesions have appeared both intracranial and extracranial, indicating progressive disease; the best response assessment for this patient was progressive disease. ^b^Patient 005, more than 30% regression of the intracranial lesion but the best response for this patient was stable disease. (B) Post Hoc subgroup analyses of CNS-ORR per the RANO-BM criteria. The subgroups were based on the baseline disease characteristics. BM, brain metastasis; CNS, central nervous system; CPS, combined positive score; IM, immunomodulatory; ORR, objective response rate; PR, partial response; RANO-BM, Response Assessment in Neuro-Oncology BMs.

**FIG A1.**
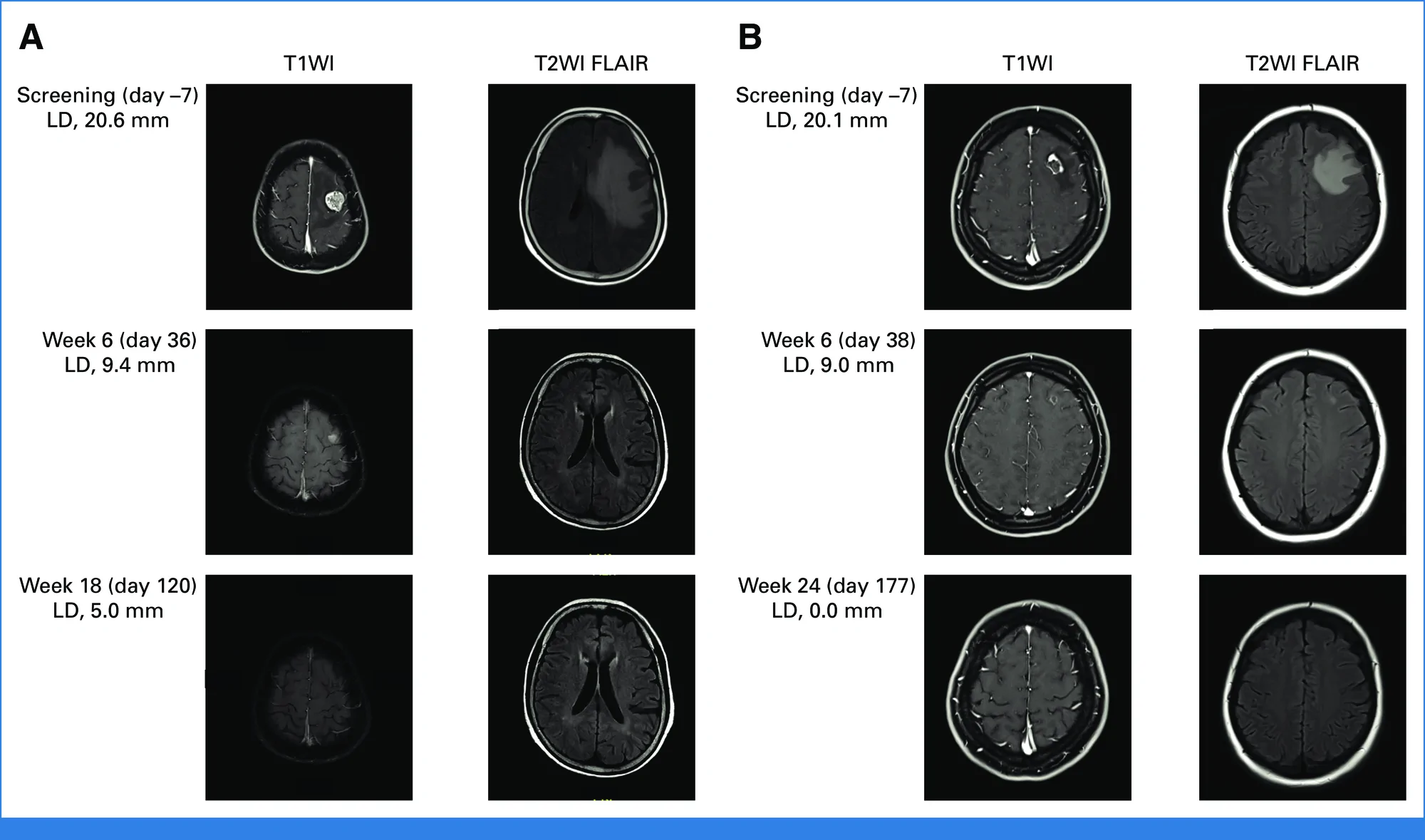
Contrast-enhanced MRI series for the patients. (A) Contrast-enhanced MRI series for the patient with a measurable intracranial lesion who had a confirmed PR. Patient 034, a 56-year-old woman with brain metastases, received second-line therapy with adebrelimab plus bevacizumab combined with cisplatin. Contrast-enhanced T1-weighted imaging and T2-weighted imaging FLAIR MRI series demonstrate a measurable target lesion with perilesional edema and midline shift at baseline. After treatment, her perilesional edema improved and the midline shift resolved. She achieved a confirmed intracranial PR with intracranial target lesions measuring 20.6 mm at baseline, reduced to 9.4 mm at week 6, and further reduced to nonmeasurable at week 18 (recorded as 5 mm per RANO-BM criteria). As of the cutoff date, the patient was still receiving treatment with an ongoing intracranial PR lasting 9.0 months. (B) Contrast-enhanced MRI series for the patient with a measurable intracranial lesion who had a confirmed CR. Patient 031, a 38-year-old woman with brain, lung, and lymph node metastases, as well as pleural effusion, received first-line therapy with adebrelimab and bevacizumab in combination with cisplatin. Contrast-enhanced T1-weighted and T2-weighted imaging FLAIR MRI series show a measurable target lesion and perilesional edema at baseline. After treatment, the perilesional edema resolved. She achieved a confirmed intracranial CR with intracranial target lesions measuring 20.1 mm at baseline, decreasing to 9.0 mm at week 6, and undetectable at week 24. As of the cutoff date, the patient was still receiving treatment and the duration of intracranial CR has reached 8.0 months. CR, complete response; LD, longest diameter; MRI, magnetic resonance imaging; PR, partial response; T1WI, T1-weighted imaging; T2WI FLAIR, T2-weighted imaging fluid-attenuated inversion recovery.

In the first stage, 10 (76.9%) of 13 patients had a CNS objective response (two CR and eight PR), and then the study proceeded to the second stage. A total of 27 patients achieved objective response in CNS, and the confirmed CNS-ORR was 77.1% (95% CI, 59.9 to 89.6), with five CRs and 22 PRs (Fig 2A). Representative MRI series illustrating intracranial responses are provided in Appendix Fig A1 (online only). Post hoc subgroup analyses demonstrated high CNS-ORR across all subgroups, such as previous CNS-directed radiation (100.0%) and PD-L1 combined positive score (CPS) ≥1 (90.9%; Fig 2B). The overall ORR was 68.6% (24/35, 95% CI, 50.7 to 83.2) and the CNS-CBR was 80.0% (28/35, 95% CI, 63.1 to 91.6) with one SD ≥24 weeks (Table 2). In addition, the triplet regimens contributed to improvement in neurologic symptoms; among the 15 patients (42.9%) with baseline neurologic symptoms, 53.3% (8/15) achieved relief (six within two cycles and two after eight cycles); the remaining seven had persistent neurologic symptoms, and none experienced worsening of their symptoms.

**TABLE 2.** Clinical Responses

End Point	All (N = 35)	95% CI
CNS objective response rate, No. (%)	27 (77.1)	59.9 to 89.6
CR	5 (14.3)	4.8 to 30.3
PR	22 (62.9)	44.9 to 78.5
SD	6 (17.1)	6.6 to 33.7
PD	2 (5.7)	0.7 to 19.2
Overall objective response rate, No. (%)	24 (68.6)	50.7 to 83.2
CR	1 (2.9)	0.1 to 14.9
PR	23 (65.7)	47.8 to 80.9
SD	9 (25.7)	12.5 to 43.3
PD	2 (5.7)	0.7 to 19.2
CNS clinical benefit rate, No. (%)	28 (80.0)	63.1 to 91.6
CR	5 (14.3)	4.8 to 30.3
PR	22 (62.9)	44.9 to 78.5
SD ≥24 weeks	1 (2.9)	0.1 to 14.9

NOTE. Evaluation of intracranial lesions was based on the RANO-BM criteria, whereas extracranial lesions were assessed according to the RECIST, 1.1.

Abbreviations: CR, complete response; PD, progressive disease; PR, partial response; RANO-BM, Response Assessment in Neuro-Oncology Brain Metastases; SD, stable disease.

Among 28 patients with disease progression, progression was intracranial-only in 32.1% (9/28) patients, extracranial-only in 35.7% (10/28) patients, and both in 32.1% (9/28) patients (Appendix Table A1). Of these 18 patients with intracranial progression, 61.1% (11/18) subsequently received intracranial radiotherapy.

**FIG 3.**
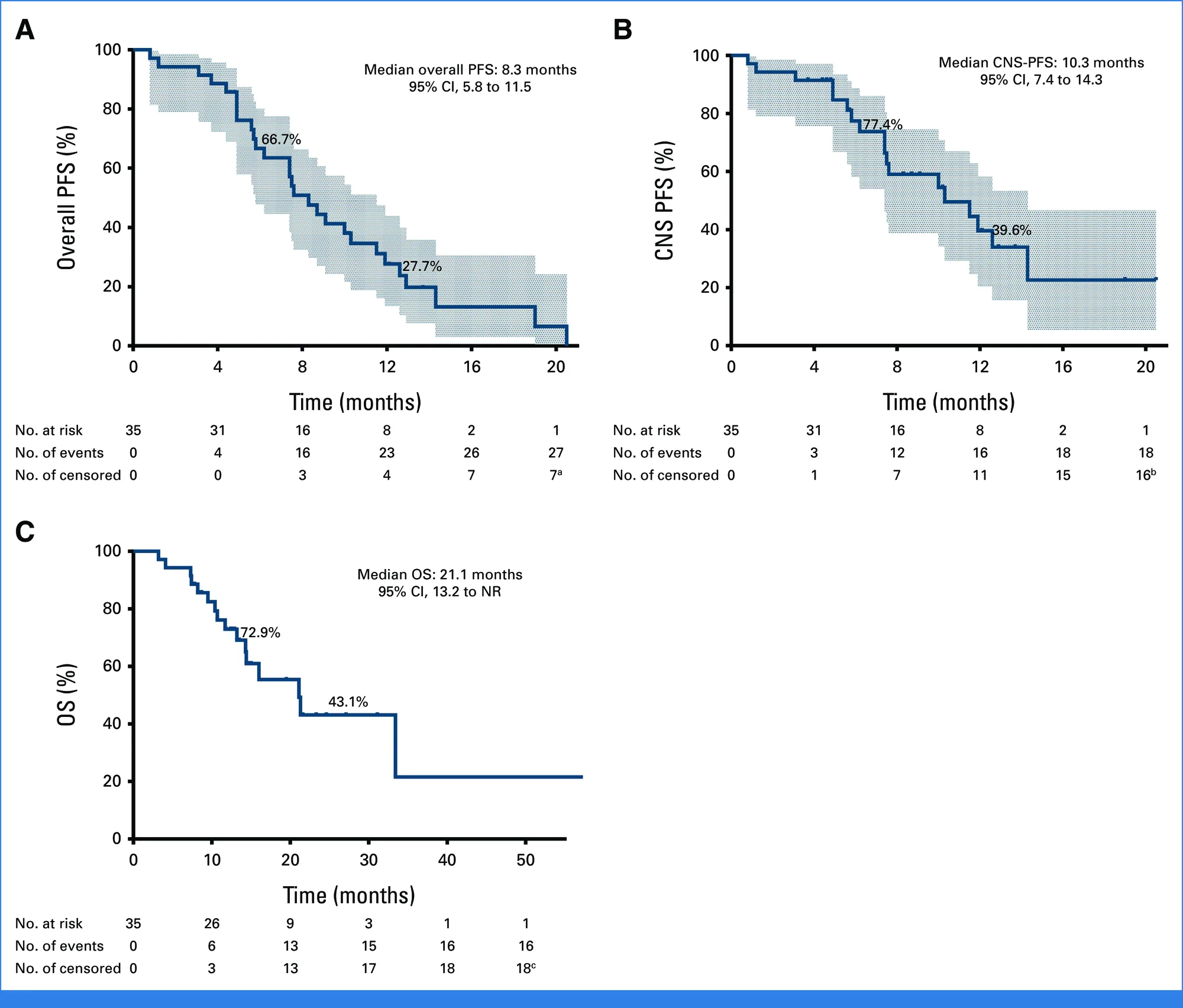
PFS and OS. (A) Kaplan-Meier estimates of overall PFS according to RANO-BM criteria and RECIST 1.1 criteria. ^a^Seven patients were censored, including three who withdrew and four who remained in the study. (B) Kaplan-Meier estimates of CNS-PFS according to RANO-BM criteria. ^b^Sixteen patients were censored, including three who withdrew, four who remained in the study, and nine who experienced only extracranial progression. (C) Kaplan-Meier estimates of OS. ^c^Eighteen patients were censored, including four who were lost to follow-up and 14 who were alive. Dotted lines indicate 95% CI. BM, brain metastasis; NR, not reached; OS, overall survival; PFS, progression-free survival.

With a median follow-up of 19.5 months (IQR, 10.9-28.1), the median overall PFS was 8.3 months (95% CI, 5.8 to 11.5, Fig 3A), whereas the median CNS-PFS was 10.3 months (95% CI, 7.4 to 14.3, Fig 3B) and the median OS was 21.1 months (95% CI, 13.2 to not reached [NR], Fig 3C). In patients with previous CNS radiation, the median CNS-PFS was 13.1 months (95% CI, NR to NR), whereas it was 10.0 months (95% CI, 6.2 to NR) in the radiation-naïve patients. The efficacy data for patients treated with cisplatin or carboplatin are available in Appendix Table A2.

**FIG A2.**
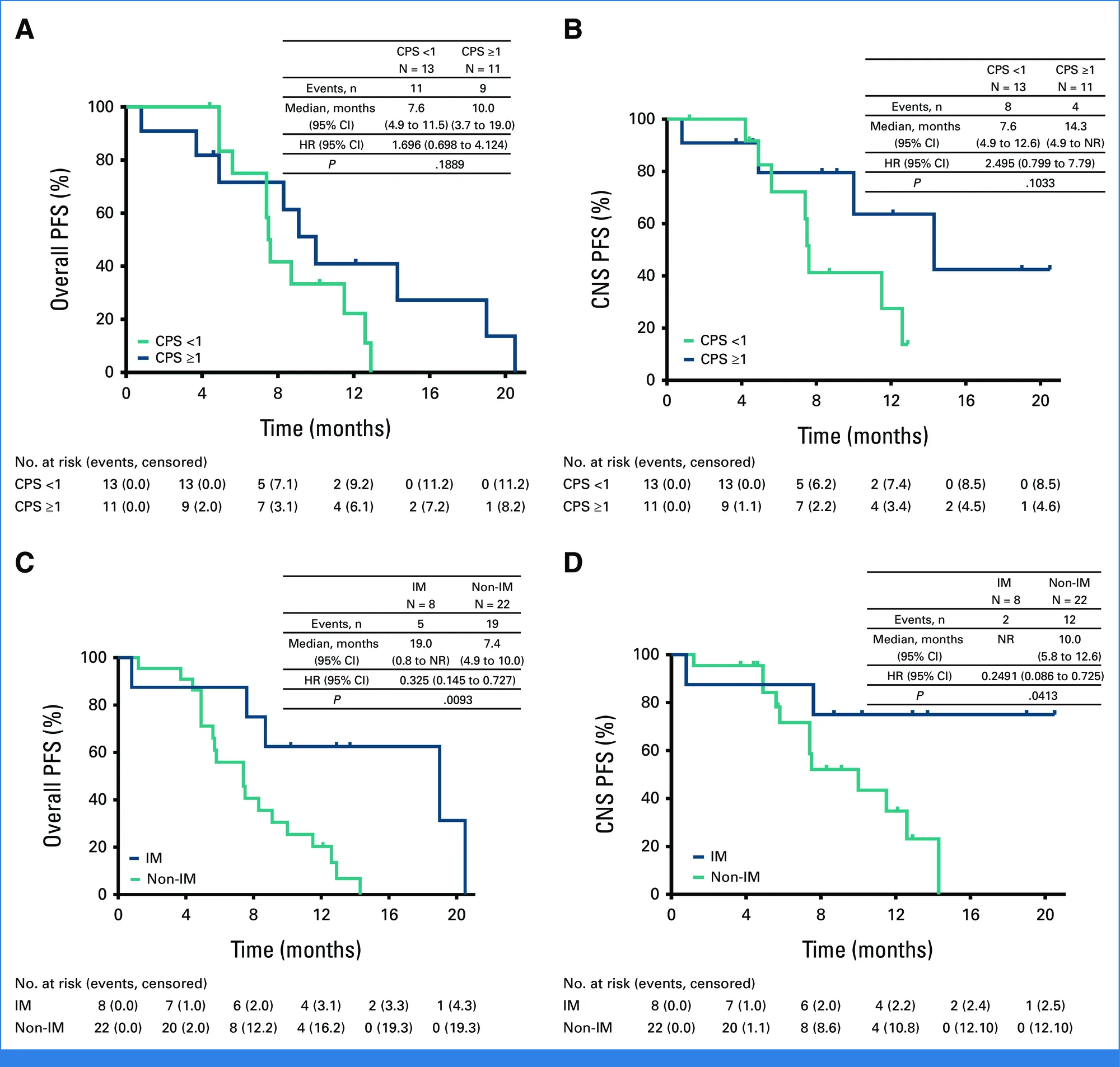
Overall PFS and CNS-PFS per Fudan TNBC classification and PD-L1 status. (A) Post hoc subgroup analysis of overall PFS based on PD-L1 status according to RANO-BM criteria and RECIST 1.1 criteria. (B) Post hoc subgroup analysis of CNS-PFS based on PD-L1 status according to RANO-BM criteria. (C) Post hoc subgroup analysis of overall PFS based on Fudan TNBC Classification according to RANO-BM criteria and RECIST 1.1 criteria. (D) Post hoc subgroup analysis of CNS-PFS based on Fudan TNBC Classification according to RANO-BM criteria. BM, brain metastasis; CPS, combined positive score; HR, hazard ratio; IM, immunomodulatory; NR, not reached; PFS, progression-free survival; OS, overall survival; RANO-BM, Response Assessment in Neuro-Oncology Brain Metastases; TNBC, triple-negative breast cancer.

In post hoc analyses, tumor samples from 30 patients (primary sites, n = 16; metastatic sites, n = 14; including four recently obtained samples at baseline) were collected for analysis of PD-L1 and Fudan TNBC classification. Although all samples underwent Fudan TNBC classification analysis, PD-L1 analysis was limited to 24 cases due to insufficient tissue availability. The analysis revealed that both PD-L1 CPS ≥1 and IM subtype showed trends toward improved PFS and CNS-PFS outcomes. In the PD-L1 status subgroup, the median overall PFS and CNS-PFS in patients with PD-L1 CPS ≥1 were 10.0 months (95% CI, 3.7 to 19.0) and 14.3 months (95% CI, 4.9 to NR), respectively, whereas those with PD-L1 CPS <1 were 7.6 months (95% CI, 4.9 to 11.5) and 7.6 months (95% CI, 4.9 to 12.6; Appendix Figs A2A and A2B), respectively. In the Fudan TNBC classification subgroup, the median overall PFS and CNS-PFS in patients with IM subtype were 19.0 months (95% CI, 0.8 to NR) and NR, respectively, whereas those with non-IM subtype were 7.4 months (95% CI, 4.9 to 10.0) and 10.0 months (95% CI, 5.8 to 12.6; Appendix Figs A2C and A2D), respectively.

### Safety

In the safety population of 35 patients, 100% (35/35) patients experienced at least one treatment-related adverse event (TRAEs; Table 3). The most common AEs were anemia (80.0%), hypomagnesaemia (74.3%), neutropenia (71.4%), and asthenia (62.9%). Grade ≥3 TRAEs occurred in 65.7% (23/35) patients, with the most common AEs being thrombocytopenia (11.4%). Serious adverse events (SAEs) related to treatment were reported in five patients (14.3%): one had grade 2 facial nerve disorder, one had grade 4 anaphylactic shock (possibly related to bevacizumab), one had grade 4 thrombocytopenia, one had grade 3 vomiting, and one had grade 3 anemia. No treatment-related deaths were reported.

**TABLE 3.** Treatment-Related Adverse Events

Eligible Patients (N = 35)	All Grade, No. (%)	Grade 1, No. (%)	Grade 2, No. (%)	Grade 3, No. (%)	Grade 4, No. (%)
TRAE	35 (100)	2 (5.7)	10 (28.6)	19 (54.3)	4 (11.4)
Hematologic	32 (91.4)	11 (31.4)	13 (37.1)	7 (20.0)	1 (2.9)
Anemia	28 (80.0)	13 (37.1)	13 (37.1)	2 (5.7)	0 (0)
Neutropenia	25 (71.4)	13 (37.1)	9 (25.7)	3 (8.6)	0 (0)
Thrombocytopenia	14 (40.0)	7 (20.0)	3 (8.6)	3 (8.6)	1 (2.9)
Nonhematologic	35 (100)	3 (8.6)	12 (34.3)	17 (48.6)	3 (8.6)
Hypomagnesaemia	26 (74.3)	23 (65.7)	2 (5.7)	1 (2.9)	0 (0)
Asthenia	22 (62.9)	9 (25.7)	11 (31.4)	2 (5.7)	0 (0)
Blood corticotrophin increased	20 (57.1)	20 (57.1)	0 (0)	0 (0)	0 (0)
Nausea	18 (51.4)	12 (34.3)	4 (11.4)	2 (5.7)	0 (0)
Decreased appetite	17 (48.6)	10 (28.6)	6 (17.1)	1 (2.9)	0 (0)
Peripheral sensory neuropathy	17 (48.6)	7 (20.0)	7 (20.0)	3 (8.6)	0 (0)
Electrocardiogram T wave abnormal	16 (45.7)	16 (45.7)	0 (0)	0 (0)	0 (0)
Sinus tachycardia	14 (40.0)	13 (37.1)	1 (2.9)	0 (0)	0 (0)
Creatinine increased	13 (37.1)	11 (31.4)	2 (5.7)	0 (0)	0 (0)
Hyperuricemia	13 (37.1)	13 (37.1)	0 (0)	0 (0)	0 (0)
Hyponatremia	13 (37.1)	12 (34.3)	0 (0)	0 (0)	1 (2.9)
Vomiting	12 (34.3)	7 (20.0)	4 (11.4)	1 (2.9)	0 (0)
Hyperglycemia	10 (28.6)	9 (25.7)	1 (2.9)	0 (0)	0 (0)
ALP increased	9 (25.7)	9 (25.7)	0 (0)	0 (0)	0 (0)
Proteinuria	9 (25.7)	4 (11.4)	5 (14.3)	0 (0)	0 (0)
ALT increased	9 (25.7)	7 (20.0)	0 (0)	2 (5.7)	0 (0)
Dizziness	9 (25.7)	6 (17.1)	2 (5.7)	1 (2.9)	0 (0)
Blood thyroid-stimulating hormone increased	9 (25.7)	8 (22.9)	1 (2.9)	0 (0)	0 (0)
Blood aldosterone increased	8 (22.9)	8 (22.9)	0 (0)	0 (0)	0 (0)
Toothache	8 (22.9)	3 (8.6)	4 (11.4)	1 (2.9)	0 (0)
Hypophosphatemia	8 (22.9)	8 (22.9)	0 (0)	0 (0)	0 (0)
Hypertension	8 (22.9)	0 (0)	5 (14.3)	3 (8.6)	0 (0)
AST increased	8 (22.9)	6 (17.1)	0 (0)	2 (5.7)	0 (0)
Hypokalemia	7 (20.0)	6 (17.1)	0 (0)	0 (0)	1 (2.9)
Headache	7 (20.0)	5 (14.3)	2 (5.7)	0 (0)	0 (0)
Potential immune-related AEs	6 (17.1)	4 (11.4)	2 (5.7)	0 (0)	0 (0)
Hyperthyroidism	4 (11.4)	4 (11.4)	0 (0)	0 (0)	0 (0)
Hypothyroidism	2 (5.7)	1 (2.9)	1 (2.9)	0 (0)	0 (0)
Hypoadrenocorticism	1 (2.9)	0 (0)	1 (2.9)	0 (0)	0 (0)

NOTE. The table shows AEs of all grades occurring in ≥20% of the safety population, and data are presented as No. (%). Potential immune-related AEs show AEs of all grades in the entire safety population, and data are presented as No. (%).

Abbreviations: AE, adverse event; TRAEs, treatment-related adverse events.

Adebrelimab treatment interruptions or discontinuations occurred in 14.3% (5/35) of patients. Bevacizumab treatment discontinuations occurred in 8.6% (3/35) of patients. Patients received a median of eight cycles (range, 1-16) of chemotherapy; cisplatin/carboplatin dose reductions, interruptions, or discontinuations occurred in 71.4% (25/35) of patients. Cisplatin/carboplatin dose reduction occurred in 12 (34.3%) of 35 patients, among which four patients (11.4%) had dose reduction before six cycle and eight patients (22.9%) had dose reduction after six cycles. Additionally, cisplatin/carboplatin discontinuations occurred in 21 patients (60.0%), with 19 patients (54.3%) discontinuing after six cycles. Among these, 11 patients (31.4%) discontinued due to AEs (Appendix Table A2).

## DISCUSSION

To our knowledge, this is the first regimen that demonstrated high intracranial activity and prolonged overall PFS/CNS-PFS in TNBC with BMs. This combination treatment achieved a confirmed CNS-ORR of 77.1% (95% CI, 59.9 to 89.6), with a median overall PFS of 8.3 months (95% CI, 5.8 to 11.5) and a median CNS-PFS of 10.3 months (95% CI, 7.4 to 14.3). Our findings demonstrate the therapeutic potential of the combination of adebrelimab, bevacizumab, and cisplatin/carboplatin in the treatment of patients with TNBC with BMs.

Our study achieved a CNS-ORR of 77.1%, higher than the CNS-ORR of 55% (per RECIST v1.1) observed in 20 patients with TNBC BM treated with utidelone plus bevacizumab in the U-BOMB trial, as well as the 37.5% intracranial response rate (evaluated per RANO-BM) reported for eight patients with TNBC BM receiving datopotamab deruxtecan in the TUXEDO-2 trial.^29,30^ The DEBBRAH study and DESTINY-Breast04 reported CNS-ORRs of 41.7% (5/12) per RANO-BM criteria and 25% (6/24) per RECIST 1.1 with T-DXd, respectively, but these HER2-low patients included only a few patients with TNBC BM.^11,31^ Furthermore, in a retrospective cohort comprising patients with TNBC BM treated with capecitabine, the reported CNS-ORR was 61%, which also remains lower than that observed in our study.^32^ A phase II trial of bevacizumab plus carboplatin in patients with active BCBM reported a CNS-ORR of 47% by RANO-BM among 38 patients (including three TNBC).^19^ Although the CNS-ORR observed with our triplet regimen is numerically higher than that reported for the doublet regimen, a direct comparison is limited by the small number of patients with TNBC. A randomized controlled trial is necessary to definitively compare the efficacy of the triplet versus the doublet regimen. Additionally, our regimen achieved a CNS-PFS of 10.3 months, comparable with outcomes observed in HER2-negative populations, including a CNS-PFS of 10.6 months reported in the U-BOMB trial, 9.7 months in the DESTINY-Breast04 study, and 4.1 months in the Daisy study with T-DXd.^11,33-35^ These findings suggest that our therapeutic regimen exhibits meaningful intracranial antitumor activity in patients with TNBC BMs. However, these trials enrolled only a restricted number of TNBC BMs, and given the differences in study designs, cross-trial comparisons should be interpreted with caution.

As for the result of survival, the median overall PFS and OS were 8.3 months and 21.1 months in our study, demonstrating a survival benefit compared with historical data for metastatic triple‑negative breast cancer (mTNBC). In the ASCENT trial, which evaluated SG in patients with mTNBC who had received two or more previous lines of therapy, the overall population achieved a median PFS of 5.6 months and a median OS of 12.1 months; in the subgroup with baseline stable BMs, the median PFS and OS were 2.8 months and 6.8 months, respectively.^12,36^ Similarly, in the phase III OptiTROP-Breast01 trial, which assessed sacituzumab tirumotecan in patients with locally recurrent or metastatic TNBC who had received two or more previous lines of therapy, the median PFS was 6.7 months, with OS not yet reached at the time of reporting.^13^ Previous studies have reported that patients with mTNBC BMs have a shorter OS compared with those with overall mTNBC.^4^ However, in our study, the PFS and OS of patients with TNBC with BMs were both longer than those of patients with mTNBC treated with SG. These results suggest this regimen may confer survival benefits in patients with TNBC with BMs. Nevertheless, it is important to note that the median line of therapy for the enrolled patients with TNBC with BMs in our study was 2 (range, 0-4), whereas the median line of therapy was 3 in both the ASCENT trial and the OptiTROP-Breast01 trial. In addition, previous exposure to immunotherapy may also influence PFS; ASCENT and OptiTROP-Breast01 included 29% and 24.6% of patients with previous immunotherapy, respectively. The ASCENT trial reported a median PFS of 4.2 months for patients with previous use of immunotherapy, compared with 6.2 months in those without previous exposure. Similarly, the OptiTROP-Breast01 study found PFS of 5.6 months versus 7.2 months, respectively. In our study, patients with previous immunotherapy were excluded. This should be taken into consideration when interpreting our findings. Furthermore, the combination of chemotherapy, immunotherapy, and bevacizumab not only exerts antitumor effects through distinct mechanisms, but may also enhance antitumor efficacy through synergistic effects. Angiogenesis and immune suppression are two critical characteristics of the tumor microenvironment. The concurrent use of antiangiogenic therapies and immunotherapy can facilitate the normalization of the tumor microenvironment by leveraging vascular and immune modulation to prolong PFS.^37^ Nonetheless, the specific mechanisms underlying these synergistic effects warrant further investigation.

Post hoc subgroup analyses showed that patients with PD-L1 CPS ≥1 (n = 11) achieved a median overall PFS of 10.0 months, which numerically exceeded the PFS of 7.6 months reported in the KEYNOTE-355 study for patients with PD-L1 CPS ≥1 advanced TNBC receiving immunotherapy plus chemotherapy.^38^ Additionally, analysis of the Fudan TNBC classification revealed that patients with the IM subtype (n = 8) experienced particularly favorable outcomes, with a median PFS of 19.0 months. These preliminary findings suggest that both PD-L1 CPS ≥1 status and IM subtype may serve as predictive biomarkers for our therapeutic regimen. However, given the limited sample sizes in these subgroup analyses, further investigation into expanded patient populations is required to substantiate their predictive utility.

The triplet regimen exhibited a manageable tolerability profile, and no new safety signals were observed and no patients experienced intracranial hemorrhage AEs and treatment-related deaths. The incidence of grade ≥3 TRAEs was 65.7%, and SAEs related to treatment were reported in 14.3% of patients. This frequency of grade ≥3 TRAEs is lower than previously reported in SCLC treated with adebrelimab plus chemotherapy regimens but consistent with rates reported in other pivotal trials in mTNBC (eg, 64% in ASCENT, 77.9% in KEYNOTE-355, 62% in IMPASSION-132, 63.1% in OptiTROP-Breast01).^12,13,21,38,39^ The most common grade ≥3 TRAEs were hematologic toxicities (22.9%), including thrombocytopenia (11.4%), neutropenia (8.6%), and anemia (5.7%), all of which were manageable with dose modifications and appropriate supportive care. Platinum-related AEs led to dose reductions in 34.3% (12/35) of patients, treatment interruptions in 11.4% (4/35), and discontinuation in 31.4% (11/35). Unlike other studies that used ≤6 cycles of chemotherapy exposure, our study reported a median exposure of eight cycles (range, 1-16). Dose reductions or discontinuations mostly occurred after cycle 6, and prolonged exposure was associated with an increased risk of platinum-related AEs.^40-42^ However, dose reductions of cisplatin/carboplatin due to AEs did not appear to adversely affect treatment efficacy. Nevertheless, caution is warranted given the limited sample size. Moreover, the incidence of chemotherapy-induced peripheral sensory neuropathy occurred in 48.6% of patients, falling within the expected range of 19%-85% reported in previous studies.^43^

This study has several limitations. First, as a phase II single-arm study without control arm, it is difficult to assess how much better (or worse) these rates are compared with published therapies. Second, although neurologic symptoms were monitored as AEs, formal quality of life and neurocognitive function assessments were not predefined in the study protocol, which limits a comprehensive evaluation of patient-centered outcomes. Finally, the subgroup analyses based on PD-L1 expression and Fudan TNBC classification should be interpreted with caution due to the use of primary or extracranial tumor tissue specimens rather than matched brain metastasis samples.

In conclusion, the results of this nonrandomized trial suggest that the triplet treatment of adebrelimab, bevacizumab, and cisplatin/carboplatin demonstrated manageable tolerability and is associated with a promising intracranial antitumor activity as well as prolonged intracranial survival benefits in TNBC with BMs. PD-L1 status and Fudan TNBC classification may predict efficacy of this triplet therapy. This combination warrants validation in randomized trials.

## Data Availability

The raw clinical and imaging data are protected due to patient privacy laws and will not be shared. All relevant de-identified data supporting the findings of this study are included within the manuscript and its Appendix files.

## APPENDIX

**TABLE A1.** The Site of First Progression

The Site of First Progression	Patients of Disease Progression (N = 28)
Intracranial alone, No. (%)	9 (32.1)
Extracranial alone, No. (%)	10 (35.7)
Lung	4 (14.3)
Lymph node	3 (10.7)
Bone	2 (7.7)
Breast	1 (3.6)
Malignant pleural effusion	1 (3.6)
Both intracranial and extracranial, No. (%)	9 (32.1)

**TABLE A2.** The Choice/Efficacy of Cisplatin/Carboplatin and Dose Reductions, Interruption, or Discontinuations of Cisplatin/Carboplatin

The Choice of Cisplatin/Carboplatin	Result
Cisplatin, No. (%)	30 (85.7)
CNS-ORR, No. (%)	23 (76.7)
CNS-PFS, months (95% CI)	11.5 (5.8 to NR)
Overall PFS, months (95% CI)	8.7 (5.8 to 11.5)
Carboplatin, No. (%)	5 (14.3)
CNS-ORR, No. (%)	4 (80.0)
CNS-PFS, months (95% CI)	7.6 (NR to NR)
Overall PFS, months (95% CI)	6.3 (NR to NR)
Cycles of treatment, median (range)	8 (1-16)
Dose reductions, interruptions, or discontinuations, No. (%)	25 (71.4)
Dose reductions, No. (%)	12 (34.3)
CNS-ORR, No. (%)	11 (91.7)
CNS-PFS, months (95% CI)	11.5 (4.9 to NR)
Overall PFS, months (95% CI)	10.2 (4.9 to 19.0)
Interruptions, No. (%)	4 (11.4)
Discontinuation reasons, No. (%)	21 (60.0)
AE	11 (31.4)
Patient's decision	9 (25.7)
Physician's decision	1 (2.9)
Progressive disease, No. (%)	13 (37.1)

Abbreviations: AE, adverse event; CNS, central nervous system; NR, not reached; ORR, objective response rate; PFS, progression-free survival.
