# Supplementary material for: Phase II Clinical Study of Adebrelimab and Bevacizumab Combined With Cisplatin/Carboplatin in Patients With Triple-Negative Breast Cancer With Brain Metastases (ABC Study)
Source: J Clin Oncol. 2026 Mar 4;44(26):2543–53. doi: 10.1200/JCO-25-02021 (PMC13557479; doi:10.1200/JCO-25-02021)
Supplement: Supplementary file 1 [file jco-44-2543-s001.pdf]

A Phase II Clinical Study of Adebrelimab and Bevacizumab Combined with Cisplatin/Carboplatin in Triple-Negative Breast Cancer Patients with Brain Metastases

Study Protocol

Protocol Number: ABC/BCBM-001/HR-BLTN-015

Version and Date: Version 2.0/13 May 2024

Principal Investigator： Professor Jian Zhang

Research Unit: Fudan University Shanghai Cancer Center

Signature

As the principal investigator of this research project, I will adhere to the ethical review measures for biomedical research involving humans by the Ministry of Health (2016), the WMA Declaration of Helsinki (2013), the CIOMS International Ethical Guidelines for Biomedical Research Involving Human Subjects (2002), and the ethical principles of Good Clinical Practice (GCP). Guided by these principles, I will conduct the study according to the protocol approved by the ethics committee, ensuring both scientific integrity and protection for the health and rights of all subjects involved.

Research Unit: Fudan University Shanghai Cancer Center

____________________	____________________	____________________
Principal Investigator (Print)	Principal Investigator (Signature)	Date (YYYY/MM/DD)

TABLE OF CONTENT

PROTOCOL SYNOPSIS 1
FLOW CHART 5
ABBREVIATIONS 9
1 Background 12
2 Objectives and Endpoints 13
3 Study Design 13
4 Selection and Withdrawal of Subjects 14
4.1 Inclusion Criteria 14
4.2 Exclusion Criteria 15
4.3 Withdrawal Criteria 15
4.4 Termination Criteria 16
5 Study Drugs 16
5.1 Name and Source of Investigational Drug 16
5.2 Treatment Plan 16
5.3 Management of Common Adverse Events 17
5.4 Dose Adjustment 19
5.5 Management, Dispensation, and Recovery of Study Drugs 26
5.6 Compliance with Study drugs 27
6 Concomitant medication 27
6.1 Prohibited medications 27
6.2 Medications to be used with caution 27
6.3 Permitted Concomitant Medications and Treatments 28
7 Study procedures 28
7.1 Screening Period 28
7.2 Trial period 30
7.3 End of study treatment/withdrawal 31
7.4 Follow-up period 32
8 Efficacy Evaluation 33
9 Safety Evaluation 33
9.1 Physical Examination and Vital Signs 33
9.2 Laboratory Examinations 33
9.3 Electrocardiogram 34
9.4 Echocardiography 35
9.5 Collection and Reporting of Adverse Events (AE) and Serious Adverse Events (SAE) 35
10 Research Management 41

10.1 Ethical and Informed Consent....41
10.2 Management of Study Drugs....42
10.3 Protocol Amendments....42
10.4 Monitoring....42
10.5 Quality Control and Assurance....43
10.6 Data Management....43
10.7 Protocol Violations....44
10.8 Data Retention....45
10.9 Publication....45
11 Statistic Analysis....45
11.1 Sample Size Calculation....45
11.2 Statistical Analysis Plan....45
11.2.1 Statistical Analysis Datasets....45
11.2.2 Statistical Analysis Methods....46
11.2.3 Statistical software....47
12 Subject Dropout....47
13 Forms of Research Publication....47
14 References....48
Appendix 1. Clinical staging criteria for breast cancer (8th edition AJCC breast cancer TNM staging)....49
Appendix 2. Physical Status Scoring Criteria (ECOG)....49
Appendix 3. Calculation of Creatinine Clearance Rate (Ccr)....49
Appendix 4. Adverse Reaction Evaluation Criteria (NCI-CTCAE Version 5.0, Partial Content)....50
Appendix 5. RANO-BM Efficacy Evaluation Criteria[17]....51

PROTOCOL SYNOPSIS

**Study Title**	A Phase II Clinical Study of Adebrelimab and Bevacizumab Combined with Cisplatin/Carboplatin in Triple-Negative Breast Cancer Patients with Brain Metastases
**Research Unit**	Fudan University Shanghai Cancer Center
**Principal Investigator**	Professor Jian Zhang
**Version /Date**	Version 2.0/13 May 2024
**Compounds**	Cisplatin/Carboplatin<br>Adebrelimab (SHR1316)<br>Bevacizumab
**Subjects**	Patients with triple-negative breast cancer brain metastases
**Objectives**	To explore treatment strategies for patients with triple-negative breast cancer with brain metastases: the efficacy and safety of adebrelimab and bevacizumab combined with cisplatin/carboplatin in patients with triple-negative breast cancer brain metastases
**Endpoints**	**Primary endpoint**:<br>Objective Response Rate in the CNS (CNS-ORR), according to RANO-BM criteria<br><br>**Secondary endpoints**:<br>Clinical Benefit Rate in the CNS (CNS-CBR: CR+ PR+ SD≥24 weeks), according to RANO-BM criteria;<br>Progression-Free Survival (PFS), including overall PFS (assessed by RANO-BM criteria and RECIST 1.1 criteria) and CNS-PFS (assessed by RANO-BM criteria);<br>Overall Survival (OS);<br>Site of First Progression;<br>Safety: Adverse Events (AE) according to NCI-CTC AE 5.0 standards<br><br>**Exploratory:**<br>Collect and preserve tumor samples from primary and/or metastatic lesions, preparing at least 10 slides, along with 8 ml each of anticoagulated blood and coagulated blood at baseline, after two cycles, and at the time of disease progression. This will be done for exploratory research aimed at investigating factors that may influence or predict the efficacy of adebrelimab combined with Bevacizumab and platinum-based therapy.
**Study Design**	This study is an open-label, prospective, single-arm, single-center phase 2 clinical trial.<br>Patients with triple-negative breast cancer brain metastases are intended to receive treatment with adebrelimab and bevacizumab combined with cisplatin/ carboplatin until disease progression, unacceptable toxicity, withdrawal of informed consent, or

	discontinuation of the study as determined by investigators. Additionally, data regarding the efficacy and safety of the drugs will be collected.<br>Disease progression is defined as intracranial lesion progression (assessed according to RANO-BM criteria) and/or extracranial lesion progression (assessed according to RECIST criteria).<br>After the termination of treatment, the physician will provide subsequent treatment recommendations based on the patient's condition.<br>Subjects who terminate treatment enter the follow-up period: 1) Safety follow-up continues until 28 days after the last administration of the investigational drug; 2) Subjects who terminate medication for reasons other than disease progression or death need to undergo efficacy follow-up until disease progression, commencement of other anti-tumor treatments, or death, whichever occurs first; 3) All subjects will undergo survival follow-up until death or until the OS data collection is completed, whichever occurs first.<br>The evaluation of intracranial ORR is based on the RANO-BM criteria.<br>The evaluation of tumor imaging for extracranial lesions is based on the RECIST 1.1 criteria.<br>The schema of study design is as follows:<br>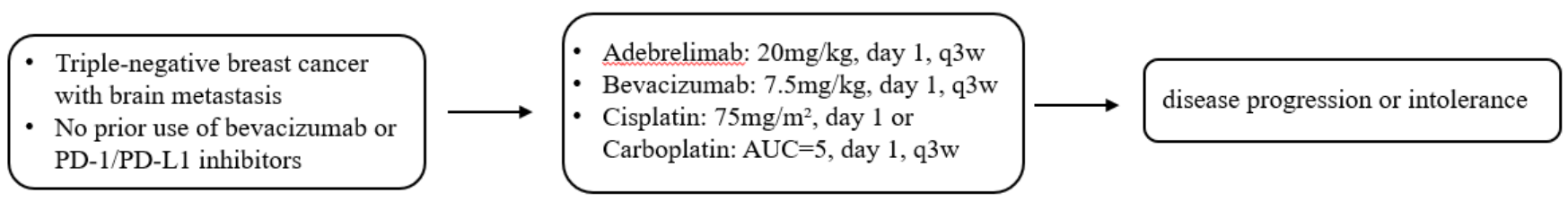
**Inclusion criteria**	1. Age ≥ 18 years old, and ≤ 70 years old, both genders;<br>2. ECOG performance status 0-2;<br>3. Pathologically confirmed HR-negative/HER2-negative breast cancer with local recurrence/metastasis; not amenable to curative surgery/radiotherapy；HR negative is defined as: ER-negative and PR-negative, the proportion of positively stained tumor cells in all tumor cells is <1%；HER2- negative is defined as: histologically confirmed to be HER2 IHC (0) or HER2 IHC (1+) or HER2 IHC (2+) and FISH (-);<br>4. It is required that no platinum drugs have been used before, or platinum drugs have been used (cisplatin/carboplatin only one regimen) and platinum-sensitive disease, defined as no progression within 4 cycles of platinum and a treatment-free interval more than 3 months before progression；<br>5. MRI confirmed brain metastases, untreated or progressive brain metastases and at least one measurable intracranial lesion with a longest diameter of 1.0 cm；<br>6. Mannitol or steroid hormone therapy is allowed before enrollment, but the dose of steroid hormone should be stable for at least one week;<br>7. Adequate function of major organs meets the following requirements:<br>(1)Blood routine ANC≥1.5×10^9/L;PLT≥75×10^9/L;Hb≥90 g/L(Allows blood transfusion

	or the use of medication to ensure that the content of hemoglobin) (2)Coagulation: INR≤1.5， APTT≤1.5×ULN, PT does not exceed the upper limit of normal;<br>(3) Blood biochemistry TBIL≤1.5×ULN; ALT and AST≤3×ULN (liver metastasis ≤5.0×ULN); Urea nitrogen ≤1.5×ULN; Cr≤1.5×ULN or creatinine clearance≥50 mL/min (Cockcroft-Gault formula);<br>(4) Cardiac ultrasound: LVEF≥50%;<br>(5)12-lead ECG: females QTcF interval <470ms and males <450ms;<br>8. Willing to join the study, sign informed consent, have good compliance and cooperate with follow-up.
**Exclusion criteria**	1. Leptomeningeal or cystic metastases confirmed by MRI or lumbar puncture.<br>2. Presence of third interstitial fluid that cannot be controlled by drainage or other methods (e.g., a large amount of pleural effusion and ascites);<br>3. Whole brain radiotherapy, chemotherapy or surgery within 14 days prior to enrollment. Has received prior therapy with Targeted therapy or Endocrine therapy within the previous week；<br>4. Subjects with HR-/HER2- who has received prior therapy with bevacizumab or PD-1/PD-L1；<br>5. Participation in any other clinical trials within 2 weeks of enrollment；<br>6. Concurrent use of any other Anti-cancer drugs；<br>7. Other malignancies within 5 years, except cured in-situ of uterine cervix carcinoma , skin basal cell carcinoma and squamous-cell carcinoma；<br>8. History of heart disease: (1) Arrhythmias requiring medical treatment or clinical significance, (2) Myocardial infarction, (3) Heart failure, (4) Any heart diseases that investigator believes not suitable for this study;<br>9. History of allergy or hypersensitivity to any of the study drugs or study drug components;<br>10. History of immunodeficiency (e.g., HIV, active hepatitis B/C) or history of organ transplantation；<br>11. A clear history of neurological or mental disorders, including epilepsy or dementia；<br>12. Pregnant or breastfeeding women. Women of childbearing potential who have a positive pregnancy test or unwilling to use adequate contraception prior to enrollment and for the duration of study participation；<br>13. According to the investigator's judgment, there is a concomitant disease that seriously endangers the safety of subjects or affects the completion of the study (including but not limited to severe hypertension, severe diabetes, active infection, thyroid disease that cannot be controlled by drugs)；<br>14. Any condition which in the investigator's opinion makes the subjects unsuitable for the study participation.

**Statistic Analysis**	The sample size is estimated based on the primary endpoint CNS-ORR. Since the limited data reported at the time of ABC study design, referring to the historical data on platinum-based regimen in solid tumor BMs and iniparib-based regimen in TNBC BMs, the null hypothesis for CNS-ORR was set at 14%[1,2], the alternative hypothesis for CNS-ORR was set at 34%. With a one-sided α of 0.05 and a power of 80%, 13 subjects would be enrolled at the first stage, the trial should be discontinued if 2 or fewer respond to the treatment. If the trial continues to the second stage and additional 18 patients should be enrolled. Overall, the null hypothesis was rejected and further investigation was favored only if more than 7 responders are obtained. Accounting for a 10% dropout rate, a total of 35 subjects were required to be enrolled.<br><br>• Efficacy analysis<br>The primary endpoint is CNS-ORR, and the analysis will be conducted on both the Full Analysis Set (FAS) and Per Protocol Set (PPS), with the FAS serving as the primary analysis set. A descriptive analysis will be performed for intracranial clinical benefit rate, progression-free survival, overall survival, and the site of first progression.<br>• Safety analysis<br>Safety analysis will be conducted on the Safety Set (SS). Adverse events (AEs) will be graded using the NCI-CTC AE version 5.0. Descriptive analysis will primarily be used to summarize data on AEs, serious adverse events (SAEs), grade ≥3 AEs, grade ≥3 SAEs, drug-related AEs, drug-related SAEs, AEs leading to dose adjustments, and AEs leading to treatment termination. Laboratory test results, vital signs, electrocardiogram data, and echocardiogram data will be analyzed using conversion tables to compare baseline and post-baseline conditions.
**Study Period**	Expected December 2019~January 2026

FLOW CHART

Project \ Time	Screening Period	Trial period				Follow-up period		
		C1-C2		C3+	Withdrawal	Safety	Efficacy[21]	Survival[22]
	D-14~D-1	The day before or the day of medication	After completion of medication	The day before or the day of medication				
Sign ICF	X							
Demographics[1]	X							
Medical History[2]	X							
ECOG Status[3]	X	X		X	X			
Vital Signs[3]	X	X		X	X			
Physical Examination[4]	X	Every 2 cycles ±7 days			X			
Blood Biochemistry[5]	X	X	X	X	X			
Blood Routine[6]	X	X	X	X	X			
Virology Test[7]	X							
Thyroid Function[8]	X	Every 2 cycles ±7 days			X			
Urine Routine[9]	X	Investigators decided on tests based on actual conditions or clinical needs			X			
Stool Routine[10]	X	Investigators decided on tests based on actual conditions or clinical needs			X			
Coagulation Function[11]	X	Investigators decided on tests based on actual conditions or clinical needs			X			
Blood Immune Cell Examination[12]	Investigators decided on tests based on actual conditions or clinical needs							
Pregnancy Test[13]	X				X			
Infectious Disease Screening[14]	X							
12-Lead ECG[15]	X	X		X	X			
Echocardiography[16]	X	Every 12 weeks (±7 days)			X			
Imaging Evaluation[17]	X	Every 2 cycles ±7 days			X		X	
Drugs[18]	X	Administer according to the research medication protocol						
Drug Dispensing /Recovery	X	1 day prior to C2		X	X			
Concomitant Medication/Therapies[19]	X	X						
AEs[20]	X	X						
Time of Progression /Death	X	X				X	X	X
Subsequent Antitumor Therapy[23]						X	X	X

Note: All procedures must be completed within protocol-specified time windows. In the event of a public holiday, they may be conducted in advance. Deviations require documentation in CRF. The researcher may adjust the examination items or frequency based on the clinical condition of the subjects.

1. Demographics (initials, gender, ethnicity, marital status, date of birth, height, weight, and calculation of body surface area based on these);
2. Medical History: including past medical history and treatment history (clinical/pathological diagnosis, time of diagnosis, clinical/pathological stage, HER2/ER/PR expression status); whether surgery was performed; whether neoadjuvant therapy was administered; whether adjuvant therapy was given; whether radiotherapy was conducted; timing of progression; basis for diagnostic progression; whether late-stage systemic treatment was received; names of drugs used in adjuvant and late-stage systemic treatments, methods of administration, duration of medication, efficacy; whether other treatment methods were used (such as bisphosphonate therapy); smoking and alcohol consumption history (frequency, amount, duration); drug allergy history (drug name, allergic symptoms); previous diseases or concurrent diseases/symptoms (disease/symptom name, names of concomitant medications, dosage, methods of administration, outcomes); bowel habits (frequency).
3. Vital Signs (including body temperature, blood pressure, respiratory rate, and pulse) and ECOG: Check once during the screening period and at each visit. Investigators decided on tests based on actual conditions or clinical needs. Perform at the end of treatment or withdrawal (if not conducted within the previous 7 days);
4. Physical Examination: Includes general condition, skin and mucous membranes, lymph nodes, head and neck, chest, abdomen, musculoskeletal, neurological reflexes, respiratory system, cardiovascular system, genitourinary system, and mental status. Check during the screening and trial periods (every 2 cycles ± 7 days); Perform at the end of treatment or withdrawal (if not conducted within the previous 7 days);
5. Blood Biochemistry: including Glucose, TP, A/G, ALT, AST, ALP, γ-GT, ALB, TBIL, DBIL, IBIL, TG, CHOL, UA, BUN, Cr, K+, Na+, CL-, Ca2+; To be checked once during the screening period and one day before or on the day of each medication visit. During first 2 cycles, weekly monitoring recommended post-infusion (≥5 days). Perform at the end of treatment or withdrawal (if not conducted within the previous 7 days). If necessary, myocardial enzyme spectrum tests can be conducted as determined by the researcher based on the subject's condition; this decision includes whether to accept test results from other hospitals.
6. Blood Routine: Includes of WBC, ANC, LC, RBC, Hb, and PLT; conducted once during the screening period and one day before or on the day of each medication visit.; During first 2 cycles, weekly monitoring recommended post-infusion (≥3 days). Perform at the end of treatment or withdrawal (if not conducted within the previous 7 days). (the researcher determines whether to accept test results from other hospitals)
7. Virology Test: Test for HbsAg (quantitative), HbsAb (qualitative), HbcAb (qualitative), HbeAg (qualitative), HCV-Ab, HBV-DNA, and HCV-RNA. Hepatitis B carriers participating in the study should have antiviral treatment arranged by the researcher as appropriate, and the viral load (HBV-DNA or HCV-RNA) should be rechecked every six weeks during the study period, with additional testing as clinically indicated;
8. Thyroid Function: including serum thyroid-stimulating hormone (TSH), free triiodothyronine (FT3), and free thyroxine (FT4). If FT3 and FT4 are unavailable, T3 and T4 may be used as substitutes. During the screening period and trial period, conduct examinations every 2 cycles ± 7 days; Perform at the end of treatment or withdrawal (if not conducted within the previous 7 days).
9. Urine Routine: includes urine protein, if urine protein++ or higher, the 24-hour urine protein quantification should be assessed; Perform one assessment during the

screening phase. Throughout the trial period, the investigator will decide on the necessity of further assessments based on the specific circumstances. Perform at the end of treatment or withdrawal (if not conducted within the previous 7 days).

10. Stool Routine: includes stool characteristics and fecal occult blood. Perform one assessment during the screening phase. Throughout the trial period, the investigator will decide on the necessity of further assessments based on the specific circumstances. Perform at the end of treatment or withdrawal (if not conducted within the previous 7 days).
11. Coagulation Function: including PT, INR, APTT, and TT. Examination will be performed during the screening period and at the end of treatment or withdrawal; Throughout the trial period, the investigator will decide on the necessity of further assessments based on the specific circumstances.
12. Blood Immune Cell Examination: including absolute lymphocyte count, absolute B lymphocyte count, B lymphocyte CD19 ratio, absolute T lymphocyte count, T lymphocyte CD3 ratio, absolute Th lymphocyte count, Th lymphocyte CD4 ratio, absolute Ts lymphocyte count, Ts lymphocyte CD8 ratio, CD4/CD8 ratio, absolute natural killer cell count, natural killer cell CD56+CD16 ratio, etc. The investigator will decide on the necessity of further assessments based on the specific circumstances.
13. Pregnancy Test: Female subjects of childbearing age must undergo blood HCG testing during the screening period to exclude pregnancy. Test will be conducted during the screening period and at the end of treatment or withdrawal. The investigator will decide on the necessity of further assessments based on the specific circumstances during trial period.
14. Infectious Disease Screening: testing for Hepatitis B surface antigen, HIV antibodies, and HCV antibodies during the screening period.
15. 12-Lead ECG: Pay attention to QT, QTc, and P-R intervals. Tests conducted before or on the day of each visit during the screening period and before medication. Perform at the end of treatment or withdrawal (if not conducted within the previous 7 days). During trial period, if the QTc interval increases by >30 msec from baseline, or QTc interval absolute value of ≥470 msec, two additional ECG examinations are required (at least 10 minutes apart). (the researcher determines whether to accept test results from other hospitals)
16. Echocardiography: Conducted during the screening period and every 12 weeks ± 7 days during the trial period. If symptoms such as chest pain or palpitations occur, additional examinations can be arranged as needed. Perform at the end of treatment or withdrawal (if not conducted within the previous 4 weeks).
17. Imaging Evaluation: the examinations include assessments of the chest, abdomen, pelvis, gynecological areas, and lymph nodes, which can be performed using CT (including enhanced CT) or MRI. Furthermore, researchers may add scanning sites during baseline or subsequent tumor assessments as needed clinically. Reports within 28 days prior to enrollment can be used, except for bone scans. The schedule for tumor imaging examinations during the trial period is established by the research team, commencing from the initiation of the study treatment. This schedule does not account for any pauses in administration due to toxic reactions. The specific evaluation criteria for imaging evaluation are as follows:

- During the screening period, participants must undergo at least a chest CT, CT or MRI of the lesion site, cranial MRI, and a bone scan. Bone scan results from the

past year are also acceptable. Throughout the trial and follow - up periods, the clinical physician may adjust the examination items as deemed necessary based on the actual conditions.

- During the trial period, examinations are conducted once every 2 cycles ±7 days; The researcher can increase the frequency of examinations according to the actual clinical needs.
- First PR/CR must be confirmed ≥4 weeks later.
- An examination is conducted at the end of treatment or withdrawal (if not conducted within the previous 8 weeks).

18. Drugs: Adebrelimab (SHR-1316) 20 mg/kg, d1, q3w; Bevacizumab 7.5 mg/kg, d1, q3w; Cisplatin 75 mg/m^2, or Carboplatin AUC=5, d1, q3w. Dosage can be adjusted according to the protocol. Treatment until disease progression, unacceptable toxicity, withdrawal of informed consent, or discontinuation of the study as determined by investigators.
19. Concomitant Medication/Therapies: Record concurrent medication within 28 days prior to enrollment and during the trial period. Once the trial treatment is discontinued, only concomitant medications and treatments used for new or unresolved adverse events related to the trial treatment should be recorded.
20. AEs: Monitoring starts on the day the ICF is signed and lasts for at least 28 days after the last treatment. AEs, concurrent medications/treatments, and unscheduled examinations should be recorded. Follow-up should continue until the AE is resolved, the subject starts a new antitumor therapy, or the condition is deemed irreversibly stable by the researcher.
21. Efficacy Follow-up: All subjects are required to undergo efficacy follow-up. From the start of the follow-up period, they will be contacted every 12 weeks (±7 days) until the PFS data collection is completed or the subject is lost to follow-up, whichever occurs first. The details of each follow-up must be recorded and entered into the CRF.
22. Survival Follow-up: Subjects who have completed both safety and efficacy follow-ups (based on the later completion date) are required to undergo survival follow-up. Survival follow-up will be conducted every 12 weeks (±7 days) until the subject dies, lost to follow-up, or the end of OS data collection is reached, whichever occurs first. The details must be recorded and entered into the CRF.
23. Subsequent Antitumor Therapy: It is necessary to record antitumor therapies from the time subjects withdraw until the end of survival follow-up; there is no need to record medications used for other diseases.

ABBREVIATIONS

ADL	Activities of Daily Living
AE	Adverse Events
AI	Aromatase inhibitor
AJCC	American Joint Committee on Cancer
AKP/ALP	Alkaline Phosphatase
ALB	Albumin
ALT	Alanine Aminotransferase
ANC	Absolute Neutrophil Count
ASCO	American Society of Clinical Oncology
AST	Aspartate Aminotransferase
AUC	Area Under the Curve
BMI	Body Mass Index
BUN	Blood Urea Nitrogen
CA125	Carbohydrate Antigen 125
CAP	College of American Pathologists
CBR	Clinical Benefit Rate
CEP17	Chromosome 17 Enumeration Probe
CD-ROM	Compact Disc Read-Only Memory
CHOL	Cholestenone
CI	Confidence Interval
Cmax	Peak Concentration
CFDA	China Food and Drug Administration
CL/F	Apparent Oral Clearance
Cr	Creatinine
CR	Complete Response
CRC	Clinical Research Coordinator
CT	Computed Tomography
CTC	Common Terminology Criteria
CDK4/6	Cyclin-Dependent Kinase 4/6
CYP	Cytochrome
DBIL	Direct Bilirubin
DFS	Disease-Free Survival
DLT	Dose-Limited Toxicity
DNA	Deoxyribonucleic Acid
DPD	Dihydropyrimidine Dehydrogenase
EC	Ethics Committee
ECG	Electrocardiogram
ECOG	Eastern Cooperative Oncology Group
eCRF	Electronic Case Report Form
EDC	Electronic Data Capture System
EGF	Epidermal Growth Factor
EGFR	Epidermal Growth Factor Receptor
ER	Estrogen Receptor
FAS	Full Analysis Set
FDG	2-Fluoro-2-deoxy-D-glucose
FISH	Fluorescence in situ hybridization
g	Gram
GCP	Good Clinical Practice
h	Hour
Hb	Hemoglobin
HBV	Hepatitis B virus
HCG	Human Chorionic Gonadotropin
HCV	Hepatitis C virus

HIV	Human Immunodeficiency Virus
HER2	Human Epidermal Growth Factor Receptor-2
HR	Hormone Receptor
HR+	Hormone Receptor Positive
HR-	Hormone Receptor Negative
IB	Investigator's Brochure
IBIL	Indirect Bilirubin
ICF	Informed Consent Form
IC50	50% Inhibition Concentration
IHC	Immunohistochemistry
INR	International Normalized Ratio
ISH	In Situ Hybridization
ITT	Intent to Treat
IU	International Unit
IV	Intravenous Injection
kg	Kilogram
L	Liter
LC	Lymphocyte Count
LDH	Lactate Dehydrogenase
LVEF	Left Ventricular Ejection Fraction
m	Meter
mL	Milliliter
mg	Milligram
mg/m^2	Milligram per Square Meter
min	Minute
mm	Millimeter
ms	Millisecond
MRI	Magnetic Resonance Imaging
MTD	Maximum Tolerated Dose
NCI	National Cancer Institute
NCCN	National Comprehensive Cancer Network
NE	Not Evaluate
NMPA	National Medical Products Administration
non-pCR	Did Not Achieve Pathological Complete Response
ORR	Objective Response Rate
OS	Overall Survival
pCR	Pathological Complete Response
PD	Disease Progression
PET	Positron Emission Tomography
PFS	Progression-Free Survival
PI	Principal Investigator
PK	Pharmacokinetics
PLT	Blood Platelet
PPS	Per Protocol Set
PR	Partial Response
PgR	Progesterone Receptor
PSA	Prostate-specific antigen
PT	Prothrombin time
QTc	QT interval corrected for heart rate
RBC	Red Blood Cell
RECIST	Response Evaluation Criteria in Solid Tumors
RNA	Ribonucleic acid
RTK	Receptor tyrosine kinase
SAE	Serious adverse events
SBP	Systolic Blood Pressure
SIE	Severe Infection
SD	Stable Disease
SAP	Statistical analysis plan

SS	Safety Set
T1/2	Half-life
TBIL	Total Bilirubin
TG	Triglyceride
Tmax	Peak time
TP ()	Plasma Total Protein
TTP	Time to progression
μmol	Micromole
UNL	Upper Normal Limit
VEGF	Vascular Endothelial Growth Factor
WBC	White Blood Cell
γGT	Gamma- glutamyl Transpeptidase

1 Background

Brain metastases are a common type of central nervous system malignancy, with an incidence rate four times higher than primary malignant tumors of the central nervous system. Approximately 20-40% of all patients with malignant tumors will eventually develop brain metastases. Among these, breast cancer being the second most common primary tumor, following lung cancer[3]. Currently, the incidence of brain metastases varies among different molecular subtypes of breast cancer. In all metastatic breast cancer (MBC) cases, the incidence rate of brain metastases is approximately 5-15%[4,5], with the highest rates seen in triple-negative breast cancer (TNBC) and HER-2 positive breast cancer, ranging from 30-45% and 30-55% respectively. Once brain metastasis occurs, the survival time is short and the prognosis is poor. The median overall survival (mOS) for all types of breast cancer brain metastasis is only 14 months, with the mOS for TNBC brain metastasis even less than 6 months [6]. Consequently, the exploration of effective therapeutic strategies to enhance the survival of patients with breast cancer brain metastasis is both necessary and urgent.

For TNBC, once brain metastasis leads to disease progression, the available targeted therapies and chemotherapeutic agents are very limited, and there is a lack of evidence-based medical support. Currently, the recommended chemotherapeutic drugs include platinum-based drugs[7]. Our center's study indicates that first-line platinum-based regimens can significantly prolong patients' progression-free survival (PFS) time. Consequently, platinum-based regimens have become the standard treatment for metastatic TNBC[8]. Moreover, in addition to effectively controlling extracranial lesions, previous studies have shown that platinum-based drugs, such as cisplatin and carboplatin, are effective in treating brain metastases in breast cancer patients[9,10]. Bevacizumab is a recombinant humanized immunoglobulin G1 monoclonal antibody that inhibits tumor angiogenesis, growth, and metastasis by targeting VEGF. The combination of bevacizumab and taxanes can be used for patients with HER-2 negative breast cancer[11]. Studies have shown that bevacizumab can cross the blood - brain barrier and alleviate brain edema after radiotherapy[12]. However, since most patients with brain metastases are excluded from clinical studies, the efficacy of bevacizumab in treating brain metastases of breast cancer remains unclear. Previous small-sample studies have shown that the combination of bevacizumab and platinum-based drugs is significantly effective for patients with brain metastases of breast cancer, however, this study included only 38 patients, of which only 8 were TNBC patients[13]. Therefore, exploring the combination of bevacizumab with chemotherapy, especially with platinum-based treatments, holds certain clinical significance for patients with HER-2 negative, particularly TNBC brain metastases.

PD-1/PD-L1 inhibitors have demonstrated initial efficacy in treating TNBC. The IMPASSION130 trial showed that in patients with PD-L1 positive tumors, the combination of Atezolizumab and nab-paclitaxel can extend OS by 10 months[14]. However, the efficacy of this combination in patients with brain metastases is still unclear. Research has shown that T cells can pass through the blood - brain barrier, suggesting that immunotherapy may be effective in patients with brain metastasis. Currently, multiple clinical studies are being conducted on the treatment of brain metastases using immune checkpoint inhibitors, mostly involving PD-1/PD-L1 monotherapy, or in combination with CTLA- 4 inhibitors, or with radiotherapy. Only one clinical study has explored the efficacy of the chemotherapy drug fotemustine in combination with PD-1/PD-L1[15]. In non-small cell lung cancer, compared with docetaxel, atezolizumab can extend OS in patients who have previously received treatment for brain metastases[16].

Adebrelimab (SHR-1316), an original humanized PD-L1 antibody drug developed by Jiangsu Hengrui

Pharmaceuticals Co., Ltd., had completed Phase I clinical trials in both Australia and China by May 15, 2018. In Australia, the trials finished dose escalation for the 20 mg/kg dose group, with 37 subjects receiving SHR1316 at four different dose levels (1 mg/kg, 3 mg/kg, 10 mg/kg, and 20 mg/kg). No dose-limiting toxicities (DLTs) or severe adverse events (SAEs) related to the drug were observed. Adverse events (AEs) associated with the drug included diarrhea, hyperthyroidism, headache, nausea, anorexia, fatigue, and infusion-related reactions, all of which were mild to moderate in severity and clinically manageable. The Phase I clinical trial in China, led by Professor Hu Xichun, has enrolled 6 subjects (3 in the 3 mg/kg dose group and 3 in the 10 mg/kg dose group). No DLTs or SAEs related to the drug occurred. The reported drug-related adverse events included fatigue, abdominal pain, and elevated bilirubin levels, all of which were mild to moderate and clinically manageable. Currently, a Phase III study is underway to investigate the first-line treatment of extensive small cell lung cancer with SHR1316 in combination with carboplatin and etoposide.

Therefore, this study intends to conduct a Phase II clinical trial to explore the efficacy and safety of treatment strategies for brain metastases in triple-negative breast cancer.

2 Objectives and Endpoints

**Objectives**	**Endpoints**
**Objective**	**Primary endpoint**
To explore treatment strategies for patients with triple-negative breast cancer with brain metastases: the efficacy and safety of adebrelimab and bevacizumab combined with cisplatin/carboplatin in patients with triple-negative breast cancer brain metastases	Objective Response Rate in the CNS (CNS-ORR), according to RANO-BM criteria
	**Secondary endpoints**
	Safety: Adverse Events (AE) according to NCI-CTC AE 5.0 standards<br>Efficacy indicators: Clinical Benefit Rate in the CNS (CNS CBR: CR+ PR+ SD≥24 weeks); Progression-Free Survival (PFS); Overall Survival (OS); Site of First Progression;

Table 1. Objectives and Endpoints

3 Study Design

This study is an open-label, prospective, single-arm, single-center Phase II clinical trial.

Patients with triple-negative breast cancer brain metastases are intended to receive treatment with adebrelimab and bevacizumab combined with cisplatin/carboplatin until disease progression, unacceptable toxicity, withdrawal of informed consent, or discontinuation of the study as determined by investigators. Additionally, data regarding the efficacy and safety of the drugs will be collected.

Subjects who terminate treatment enter the follow-up period: 1) Safety follow-up continues until 28 days after

the last administration of the investigational drug; 2) Subjects who terminate medication for reasons other than disease progression or death need to undergo efficacy follow-up until disease progression, commencement of other anti-tumor treatments, or death, whichever occurs first; 3) All subjects will undergo survival follow-up until death or until the OS data collection is completed, whichever occurs first.

The schema of study design is as follows:

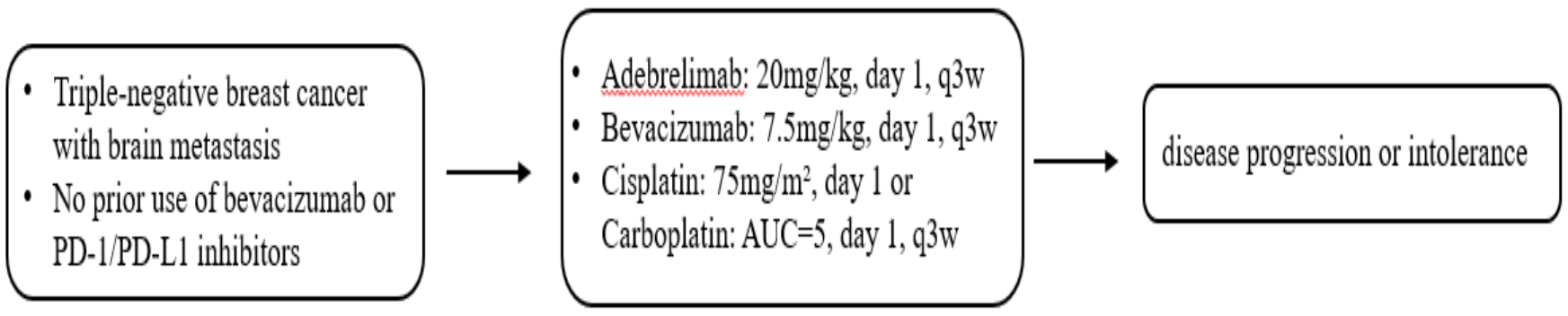

Figure 1. The Schema of Study Design

4 Selection and Withdrawal of Subjects

4.1 Inclusion Criteria

Subjects must meet all of the following eligibility criteria to be enrolled in this trial:

1. Age≥18 years old, and≤70 years old, both genders;
2. ECOG performance status 0-2;
3. Pathologically confirmed HR-negative/HER2-negative breast cancer with local recurrence/metastasis; not amenable to curative surgery/radiotherapy；HR negative is defined as: ER-negative and PR-negative, the proportion of positively stained tumor cells in all tumor cells is <1%; HER2- negative is defined as: histologically confirmed to be HER2 IHC (0) or HER2 IHC (1+) or HER2 IHC (2+) and FISH (-);
4. It is required that no platinum drugs have been used before, or platinum drugs have been used (cisplatin/carboplatin only one regimen) and platinum-sensitive disease, defined as no progression within 4 cycles of platinum and a treatment-free interval more than 3 months before progression；
5. MRI confirmed brain metastases, untreated or progressive brain metastases and at least one measurable intracranial lesion with a longest diameter of 1.0 cm；
6. Mannitol or steroid hormone therapy is allowed before enrollment, but the dose of steroid hormone should be stable for at least one week;
7. Adequate function of major organs meets the following requirements:

(1) Blood routine ANC≥1.5×10^9/L; PLT≥75×10^9/L; Hb≥90 g/L (Allows blood transfusion or the use of medication to ensure that the content of hemoglobin)

(2) Coagulation: INR≤1.5，APTT≤1.5×ULN, PT does not exceed the upper limit of normal;

(3) Blood biochemistry TBIL≤1.5×ULN; ALT and AST≤3×ULN (liver metastasis≤5.0×ULN); Urea nitrogen≤1.5×ULN; Cr≤1.5×ULN or creatinine clearance≥50 mL/min (Cockcroft-Gault formula);

(4) Cardiac ultrasound: LVEF≥50%;

(5) 12-lead ECG: females QTcF interval <470msec and males <450ms;

8. Willing to join the study, sign informed consent, have good compliance and cooperate with follow-up.

4.2 Exclusion Criteria

Subjects will not be included if any of the following conditions are present:

1. Leptomeningeal or cystic metastases confirmed by MRI or lumbar puncture.
2. Presence of third interstitial fluid that cannot be controlled by drainage or other methods (e.g., a large amount of pleural effusion and ascites);
3. Whole brain radiotherapy, chemotherapy or surgery within 14 days prior to enrollment. Has received prior therapy with Targeted therapy or Endocrine therapy within the previous week；
4. Subjects with HR-/HER2- who has received prior therapy with bevacizumab or PD-1/PD-L1；
5. Participation in any other clinical trials within 2 weeks of enrollment；
6. Concurrent use of any other Anti-cancer drugs；
7. Other malignancies within 5 years, except cured in-situ of uterine cervix carcinoma , skin basal cell carcinoma and squamous-cell carcinoma；
8. History of heart disease: (1) Arrhythmias requiring medical treatment or clinical significance, (2) Myocardial infarction, (3) Heart failure, (4) Any heart diseases that investigator believes not suitable for this study;
9. History of allergy or hypersensitivity to any of the study drugs or study drug components;
10. History of immunodeficiency (e.g., HIV, active hepatitis B/C) or history of organ transplantation；
11. A clear history of neurological or mental disorders, including epilepsy or dementia；
12. Pregnant or breastfeeding women. Women of childbearing potential who have a positive pregnancy test or unwilling to use adequate contraception prior to enrollment and for the duration of study participation；
13. According to the investigator's judgment, there is a concomitant disease that seriously endangers the safety of subjects or affects the completion of the study (including but not limited to severe hypertension, severe diabetes, active infection, thyroid disease that cannot be controlled by drugs)；
14. Any condition which in the investigator's opinion makes the subjects unsuitable for the study participation.

4.3 Withdrawal Criteria

4.3.1 Withdrawal Criteria

Withdrawal from this clinical study:

1. The subject withdraws the informed consent form;
2. The subject is found to have significantly violated the inclusion or exclusion criteria after enrollment.

Termination of treatment. However, follow-up will continue in accordance with the study requirements:

1. Disease progression as assessed by imaging;
2. Occurrence of any adverse events, laboratory abnormalities, or other medical conditions that may result in the subject no longer benefiting from continued medication;
3. Pregnancy occurring during the study period;

4. Medical or ethical reasons that affect the continuation of the study;

5. Serious violation of the trial protocol, and the investigator assesses that the treatment should be terminated;

6. Other reasons the investigator believes the study medication cannot be continued.

4.3.2 Treatment for Withdrawaer

Every effort must be made to complete the end of study treatment/withdrawal visits as per the protocol. For subjects who terminate study treatment, follow-up for safety, efficacy (if required), and survival will be performed as specified for the follow-up period. Researchers can recommend or provide new or alternative treatments to patients according to their actual conditions.

4.4 Termination Criteria

The study may be terminated or suspended prematurely if there is sufficient reason. If the study is terminated or suspended prematurely, the researcher must immediately inform the subjects, and the principal investigator must notify the clinical trial institution/ethics committee of the research center in writing, explaining the reasons for the premature termination or interruptions.

The study termination criteria include, but are not limited to, the following:

1) An unexpected, significant, or unacceptable risk to the subject is found;
2) Major errors are found in the scheme during the execution;
3) The study drug/trial treatment is ineffective, or it is not meaningful to continue the trial;
4) Completing the trial was extremely difficult due to factors such as significant delays in subject enrollment or frequent protocol deviations.

5 Study Drugs

5.1 Name and Source of Investigational Drug

The drugs involved in this project include Adebrelimab, Bevacizumab and Cisplatin/Carboplatin. Adebrelimab is produced and provided by Jiangsu Hengrui Pharmaceutical Co., Ltd. Bevacizumab and Cisplatin or Carboplatin are both commercially available drugs.

5.2 Treatment Plan

Adebrelimab：20 mg/kg，d1，q3w；

Bevacizumab: 7.5 mg/kg, day 1, q3w;

Cisplatin 75 mg/m^2, or Carboplatin AUC=5, d1, q3w.

Adebrelimab, bevacizumab, and cisplatin or carboplatin are administered sequentially on the first day of each

cycle. Adebrelimab is administered via intravenous infusion first. At the end of the infusion, the infusion line is flushed with an adequate amount of saline solution or 5% glucose solution (according to the medical standards of the research unit). Bevacizumab is then administered via intravenous infusion, and the infusion line is similarly flushed at the end. Finally, cisplatin or carboplatin is administered via intravenous infusion. For each infusion, the line is flushed with an adequate amount of saline solution or 5% glucose solution to ensure proper administration.

Medication order: Adebrelimab→Bevacizumab→Cisplatin or Carboplatin (at least 30 minutes apart)

Medication dosage may be adjusted based on the adverse reactions of the subjects, in accordance with the protocol. Subjects will continue medication until disease progression, unacceptable toxicity, withdrawal of informed consent, or discontinuation of the study as determined by investigators.

5.3 Management of Common Adverse Events

When adverse events occur during the study, researchers should actively manage symptoms based on clinical circumstances and standard clinical procedures, and record details of concomitant treatments and medications both in the medical history and in the CRF. Researchers should provide medical treatment according to clinical circumstances, while the following treatment methods are for guidance:

Adebrelimab Supportive Treatment Guidelines:

The subjects should receive supportive therapeutic measures deemed necessary and appropriate by the investigator. Supportive therapeutic measures for addressing adverse events (AEs) with potential immunological relevance are listed below, including the use of corticosteroids orally or intravenously, and the use of other anti-inflammatory drugs in cases where symptoms do not improve with corticosteroid treatment. The tapering of corticosteroids may require multiple cycles, as symptoms may worsen during the tapering process. Other potential causes that may necessitate additional supportive therapy, such as metastatic disease or bacterial or viral infections, should be ruled out as much as possible. If the researcher confirms that the AE is related to Adebrelimab, the supportive treatments listed below can be used. If it is not related to Adebrelimab, the supportive treatments listed below are not necessary.

Diarrhea/Colitis:

Carefully monitor symptoms and signs of enterocolitis (such as diarrhea, abdominal pain, bloody or mucoid stools, with or without fever) and intestinal perforation (e.g., peritonitis and intestinal obstruction). All subjects who develop diarrhea/colitis should be advised to drink an adequate amount of fluids. If adequate oral intake of fluids is not possible,

intravenous fluid and electrolyte replacement should be administered.

- For Grade 2 or higher diarrhea, gastrointestinal (GI) consultation and endoscopy should be considered to confirm or rule out colitis.
- For Grade 2 diarrhea/colitis, oral corticosteroids should be administered.
- For Grade 3 or 4 diarrhea/colitis, intravenous corticosteroid therapy should be initiated, followed by high-dose oral corticosteroids.
- After symptoms improve to Grade 1 or below, corticosteroid tapering should begin and continue for no less than 4 weeks.

Elevated AST, ALT, or bilirubin:

- For Grade 2 events, intravenous or oral corticosteroids should be administered, and liver function should be monitored more frequently until baseline values are restored (consider testing once a week).
- For Grade 3-4 events, intravenous corticosteroid therapy should be administered for 24-48 hours.
- After symptoms improve to Grade 1 or below, corticosteroid tapering should begin and continue for no less than 4 weeks.

Hyperthyroidism/Hypothyroidism:

Thyroid disorders may occur at any time during treatment. Monitor changes in thyroid function (at the start of treatment and regularly during treatment) as well as clinical symptoms and signs of thyroid disease.

- For Grade 2 hyperthyroidism, non-selective β-blockers (e.g., propranolol) are recommended as initial treatment.
- For Grade 3-4 hyperthyroidism, intravenous corticosteroids should be initiated, followed by oral corticosteroids.
- After symptoms improve to Grade 1 or below, corticosteroid tapering should begin and continue for no less than 4 weeks.
- During corticosteroid tapering, appropriate hormone replacement therapy may be required. For Grade 2-4 hypothyroidism, thyroid hormone replacement therapy (e.g., levothyroxine) may be considered.

Pneumonia:

For Grade 2 pneumonia, systemic corticosteroid therapy should be administered. After symptoms improve to Grade 1 or below, corticosteroid tapering should begin and continue for no less than 4 weeks. If long-term corticosteroid use is required, prophylactic antibiotics should be administered.

Immune-related Hypophysitis:

- For Grade 2 hypophysitis, continuous corticosteroid therapy should be administered. After symptoms improve to Grade 1 or below, corticosteroid tapering should begin and continue for no less than 4 weeks. During corticosteroid tapering, appropriate hormone replacement therapy may be required.
- For Grade 3 or 4 hypophysitis, intravenous corticosteroids should be initiated, followed by oral corticosteroids. After symptoms improve to Grade 1 or below, corticosteroid tapering should begin and continue for no less than 4 weeks. During corticosteroid tapering, appropriate hormone replacement therapy may be required.

Renal Failure or Nephritis:

- For Grade 2 events, corticosteroid therapy should be administered.
- For Grade 3-4 events, systemic corticosteroid therapy should be administered. After symptoms improve to Grade 1 or below, corticosteroid tapering should begin and continue for no less than 4 weeks.

5.4 Dose Adjustment

5.4.1 Recommendations for Dose Adjustment of Platinum-Based Drugs

The dose adjustment of Cisplatin or Carboplatin should be determined by the investigator according to clinical practice. This section is provided for the investigator's reference.

The levels of dose adjustment for chemotherapeutic drugs are shown in Table 2 below:

Table 2. Dose Adjustment Levels for Chemotherapeutic Drugs

Chemotherapeutic Drugs	Initial Dose	Reduce by One Dose Level	Reduce by Two Dose Level
Cisplatin	75 mg/m^2	60 mg/m 2	45 mg/m 2
Carboplatin	AUC=5	At 75% of the initial dose	At 50% of the initial dose

Recommended dose adjustments for chemotherapy-related hematologic toxicity are shown in Table 3 below:

Table 3 Recommended Dose Adjustments Based on Chemotherapy-Related Hematologic Toxicity from the Previous Cycle

Events Related to Chemotherapy	Cisplatin/Carboplatin
ANC<0.5×10^9/L for More Than 5 Days	Previously No Prophylactic Use of G-CSF ※, Prefer Prophylactic Use of G-CSF This Time, Otherwise Reduce by One Dose Level
Febrile neutropenia (ANC<0.5×10^9/L, A single temperature≥38.3°C or temperature≥38.0°C for	Previously No Prophylactic Use of G-CSF ※, Prefer Prophylactic Use of G-CSF This Time, Otherwise Reduce by

over 1 hour)	One Dose Level
Platelet count <50×10^9/L, accompanied by bleeding events	Reduce by One Dose Level
Platelet count <25×10^9/L	Reduce by One Dose Level
Hemoglobin <65g/L	Reduce by One Dose Level

※ Prophylactic use of G-CSF can opt for long-acting G-CSF injections. If short-acting G-CSF is chosen, it is recommended to administer at a dose of 5 μg/kg, once daily for 7 days (qd*7d).

If the aforementioned toxic reactions occur a third time after two dose reductions, chemotherapy should be discontinued.

Recommended dose adjustments for chemotherapy-related non-hematological toxicity are shown in Table 4 below:

Table 4. Recommended Dose Adjustments Based on Chemotherapy-Related Non-Hematologic Toxicity from the Previous Cycle

Adverse events	CTCAE Grade	Cisplatin/Carboplatin
Nausea or Vomiting*	Grade 3 or 4	Reduce by One Dose Level
Diarrhea*	Grade 3 or 4	Reduce by One Dose Level
Rash	Grade 3 or 4	Initial Dose
Ototoxicity	Grade 2	Pause Medication**
	Grade 3 or 4	Discontinue Medication
Neurotoxicity	Grade 2	Reduce by One Dose Level
	Grade 3 or 4	Discontinue Medication
Other Non-Hematologic Toxicities	Grade 3 or 4	Reduce by One Dose Level

*Supportive symptomatic treatment is acceptable

** If the subject develops ototoxicity, the use of cisplatin should be paused until the hearing analysis results show that auditory acuity is within the normal range.

If a decrease in creatinine clearance rate is observed, adjust the dose of carboplatin according to Table 5.

Table 5 Recommended Carboplatin Dose Adjustments Based on Creatinine Clearance Levels

Calculated creatinine clearance rate	Dose adjustment
≥50 ml/min	No adjustment is needed.
35-50 ml/min	Dosing calculated according to the Calvert formula
25-35 ml/min (including 35)	Dosing calculated according to the Calvert formula

≤25	Pause Medication

Note: Calvert formula: Carboplatin dose (mg) = Target AUC (mg/ml/min) × [Creatinine clearance (ml/min) + 25]

5.4.2 Interruptions and Resumption of Adebrelimab Medication

Based on the severity of toxicity in the subjects and the results of the risk-benefit assessment, the investigator may consider the adjustment principles described below at their discretion. Increasing or decreasing the dose of Adebrelimab is not permitted; only interruptions or termination is permitted. The maximum allowable interval for interruptions of adebrelimab is 12 weeks (calculated from the last actual administration time), otherwise, the treatment with Adebrelimab should be terminated. If the interruptions of adebrelimab exceeds 12 weeks, and the investigator believes that the subject may benefit from continued treatmen, a discussion with the participating medical personnel is required.

The time window for each administration is calculated from the first administration date. Delays generally should not exceed 3 days (subsequent cycles proceed as originally planned), but in special circumstances, with reasons noted, they may exceed 3 days. If the delay exceeds 3 days but is less than 14 days, it is recommended to recalculate the time window based on the actual administration time for this instance, while the subsequent administration time window remains 3 days. If the delay exceeds 14 days, it is recommended not to supplement the dose in this cycle and to continue medication in the next cycle.

Adverse events related to Adebrelimab may be immune toxicities, which can occur shortly after the first administration or months after the last administration. If the situations listed in Table 6 occur, Adebrelimab treatment should be delayed or interrupted. If the investigator, during the clinical operation process, considers the benefit/risk ratio for the subject and believes that the operations listed in Table 6 cannot be followed or encounters unlisted situations where Adebrelimab treatment must be delayed or resumed, a discussion with the participating medical personnel is required.

The dose adjustment for Adebrelimab are referenced in Table 6, and the management of Adebrelimab related infusion reactions is referenced in Table 7.

In situations not explicitly defined in the protocol, the investigator must consider the benefit/risk ratio for the subject before making decision. In situations explicitly defined in the protocol, treatment must be interrupted /delayed as stipulated.

Table 6: Dose Adjustment for Adebrelimab-Related Immune-Mediated Toxicity

Toxicity Related to Adebrelimab	Level of Treatment interruptions (NCI CTC v5.0)	Resuming Treatment Time	Permanent Discontinuation
Diarrhea/Colitis	Grade 2-3	Toxicity reduced to grade 0-1	Toxicity not resolved within 12 weeks after the last administration, or the corticosteroid dosage cannot be reduced to 10 mg/day or lower of prednisone (or equivalent) within 12 weeks
	Grade 4	Permanent Discontinuation	Permanent Discontinuation
Elevation of AST, ALT, or bilirubin [a]	Grade 2	Toxicity reduced to grade 0-1	Toxicity not resolved within 12 weeks after the last administration, or the corticosteroid dosage cannot be reduced to 10 mg/day or lower of prednisone (or equivalent) within 12 weeks
	Grade 3-4	Permanent discontinuation [a]	Permanent Discontinuation
Type 1 diabetes (if newly diagnosed) or Hyperglycemia with signs of pancreatic β-cell failure	Grade 3-4 or newly diagnosed type 1 diabetes	Adebrelimab treatment can be resumed after the subject's clinical and metabolic condition stabilizes.	
Hyperthyroidism	Grade 3	Toxicity reduced to grade 0-1	Toxicity not resolved within 12 weeks after the last administration, or the corticosteroid dosage cannot be reduced to 10 mg/day or lower of prednisone (or equivalent) within 12 weeks
	Grade 4	Permanent Discontinuation	Permanent Discontinuation
Hypothyroidism	Grade 2-4	After initiating thyroid hormone replacement therapy, treatment can continue.	

Pneumonia	Grade 2	Toxicity reduced to grade 0-1	Toxicity not resolved within 12 weeks after the last administration, or the corticosteroid dosage cannot be reduced to 10 mg/day or lower of prednisone (or equivalent) within 12 weeks
	Grade 3-4	Permanent Discontinuation	Permanent Discontinuation
Hypophysitis	Grade 2-4	Toxicity reduced to Grade 0-1; treatment can continue after initiating endocrine replacement therapy.	Toxicity not resolved within 12 weeks after the last administration, or the corticosteroid dosage cannot be reduced to 10 mg/day or lower of prednisone (or equivalent) within 12 weeks
Infusion reaction	Grade 2 [b]	Toxicity reduced to grade 0-1	If symptoms recur despite adequate prophylactic medication, permanently discontinue the drug.
	Grade 3-4	Permanent Discontinuation	Permanent Discontinuation
Renal failure or nephritis	Grade 2	Toxicity reduced to grade 0-1	Toxicity not resolved within 12 weeks after the last administration, or the corticosteroid dosage cannot be reduced to 10 mg/day or lower of prednisone (or equivalent) within 12 weeks
	Grade 3-4	Permanent Discontinuation	Permanent Discontinuation
Other drug-related toxicities [c]	Grade 3	Toxicity reduced to grade 0-1	Toxicity not resolved within 12 weeks after the last administration, or the corticosteroid dosage cannot be reduced to 10 mg/day or lower of prednisone (or equivalent) within 12 weeks
	Grade 4	Permanent Discontinuation	Permanent Discontinuation

Note: If any serious or ≥ Grade 3 (pneumonia ≥ Grade 2) drug-related adverse event (AE) occurs again, or any life-threatening AE, the drug should be permanently discontinued.

a) For subjects with liver metastases who have a baseline elevation of AST or ALT to Grade 2, if AST or ALT increases by ≥50% from baseline and persists for at least one week during treatment, medication should be permanently discontinued.

b) If symptoms alleviate within one hour of temporarily stopping the medication, infusion can continue at 50% of the initial infusion rate. Otherwise, medication can only be resumed after symptoms have completely resolved. Subjects should also be adequately premedicated during the next administration. For further management, please refer to section 8 on recommendations for handling infusion reactions.

c) For subjects experiencing intolerable or persistent Grade 2 drug-related adverse events (AEs), the researcher may consider temporarily suspending Adebrelimab. For subjects with persistent Grade 2 drug-related AEs, if toxicity does not reduce to Grade 0-1 within 12 weeks after the last dose, medication should be permanently discontinued.

Table 7: Treatment Recommendations for Infusion Reactions

CTCAE Grade	Clinical Symptoms	Clinical Management	Adebrelimab Treatment
Grade 1 (Mild)	Mild and transient reactions;	Observe at bedside and monitor closely until recovery. (In future, pre-medication before infusion is recommended: Diphenhydramine 50 mg, or equivalent and/or Acetaminophen 325-1000 mg, at least 30 minutes before Adebrelimab administration)	Continue
Grade 2 (Moderate)	Moderate reactions requiring treatment or interruption of medication, which can be rapidly relieved after symptomatic treatment (such as antihistamines, non-steroidal anti-inflammatory drugs, analgesics, bronchodilators, intravenous fluids, etc.)	Intravenous saline infusion, diphenhydramine 50 mg IV or equivalent and/or acetaminophen 325-1000 mg;<br>Observe at bedside and monitor closely until recovery.<br>Corticosteroids or bronchodilators may be considered as clinically needed;<br>Record the infusion amount of the investigational drug in the original medical records;<br>For future recommendations of pre-infusion prophylactic medication: at least 30 minutes before Adebrelimab administration, diphenhydramine 50 mg, or equivalent and/or acetaminophen 325-1000 mg. Cortisol hormone as needed (equivalent to a dose of 25 mg hydrocortisone).	Pause Medication. When re-administering after symptoms disappear, 50% of the initial infusion rate. If there are no complications within 30 minutes, the infusion rate can be increased to the original 100%. Monitor closely. If symptoms recur, do not infuse Adebrelimab for the current treatment.

≥ Grade 3 (Severe)	Grade 3: Severe reaction, no rapid relief after treatment and/or medication interruptions; or symptom recurrence after relief; sequelae requiring hospitalization.<br>Grade 4: Life-threatening	Immediately stop the infusion of Adebrelimab;<br>Start intravenous infusion of saline solution.<br>• Recommend bronchodilators, subcutaneous injection of 0.2-1 mg of 1:1000 epinephrine solution, or slow intravenous injection of 0.1-0.25 mg of 1:10000 epinephrine solution, if necessary, and/or 50 mg diphenhydramine with 100 mg methylprednisolone or equivalent intravenously;<br>• Follow the guidelines of the research institution for treating allergic reactions;<br>Observe at bedside and monitor closely until recovery.	Permanently discontinue.

5.4.3 Recommendations for dose adjustment of Bevacizumab

There are no recommendations for dose reduction of Bevacizumab. Discontinuation is recommended in the following situations:

1. Reversible posterior leukoencephalopathy syndrome (RPLS);
2. Grade 3/4 hemorrhagic events;
3. Arterial thromboembolism of any grade;
4. Grade 4 hypertension (hypertensive crisis);
5. Grade 4 proteinuria (nephrotic syndrome);
6. Grade 3/4 Left Ventricular Dysfunction (CHF);
7. Gastrointestinal perforation of any grade;
8. Tracheoesophageal fistula of any grade;
9. Grade 4 non-tracheoesophageal fistula;
10. Any grade of hypersensitivity/allergic reaction related to bevacizumab.

5.4.4 Administration Delay

The following conditions must be met before starting treatment in any cycle, otherwise administration needs to be delayed:

1. Absolute Neutrophil Count (ANC) ≥1.5×10^{9}/L
2. Platelet count ≥75×10^{9}/L
3. Hemoglobin ≥80g/L

If the investigator determines that the toxicity is primarily caused by one of the investigational drugs, dose reduction or interruptions of that single drug is acceptable. If it is unclear whether the toxicity is caused by two or more investigational drugs, dose reduction or interruptions of all drugs considered relevant may be implemented

simultaneously, at the investigator's discretion.

For Adebrelimab /platinum/bevacizumab, the administration window is the scheduled administration date (calculated from the first administration date) ±3 days. If the administration occurs beyond the window period (3 days), it is considered delayed administration, and subsequent administration dates should be recalculated based on the actual date of the last administration. In situations where a delay is required due to toxicity reasons (without being able to specify which medication is responsible), and recovery to the standard for re-administration is expected within 2 weeks, Adebrelimab/platinum/bevacizumab should be delayed simultaneously. Upon recovery, the entire combination regimen should be administered in the order originally specified, as much as feasible. During the combination therapy, if the delay due to the toxicity of chemotherapy/bevacizumab is expected to exceed 2 weeks, only Adebrelimab should be administered until the toxicity recovers to the standard for chemotherapy/bevacizumab administration. Subsequently, chemotherapy/bevacizumab and Adebrelimab should be restarted in a new cycle, with chemotherapy being allowed to be paused for a maximum of 9 consecutive weeks. If it exceeds 9 weeks, chemotherapy should be terminated. If the delay due to the toxicity of Adebrelimab is expected to exceed 2 weeks, chemotherapy will be administered alone until the toxicity recovers to the Adebrelimab administration standard, after which chemotherapy/bevacizumab combined with Adebrelimab will be resumed. Adebrelimab can be paused for up to 12 consecutive weeks, and if it exceeds 12 weeks, Adebrelimab will be discontinued.

5.5 Management, Dispensation, and Recovery of Study Drugs

The management, dispensation, and recovery of study drugs in this study are handled by designated personnel. The researcher must ensure that all study drugs are used only for subjects participating in this clinical trial, and dosage and usage should comply with the trial protocol. Remaining drugs should be returned to the research unit and must not be transferred to any non-clinical trial participants.

The study drugs should be stored according to the requirements of the drug instructions. The dispensation and recovery of drugs shall be conducted in accordance with GCP requirements. At the end of the study, remaining drugs and empty boxes shall be collected. Each dispensation and recovery of drugs must be promptly recorded on a dedicated record sheet.

Monitors are responsible for overseeing the supply, usage, storage, and disposal of remaining study drugs in the clinical trial.

5.6 Compliance with Study drugs

When study drugs have been distributed to individual subjects, these subjects should be required to return any unused study drugs during each subsequent visit. Subjects should record their medication information in a diary daily.

During the subject's medication period, the research unit will assess the compliance with the investigational medicinal products and dosing (e.g., taking the prescribed dose of medication every day) at each clinical visit (excluding follow-up visits). At each study visit, research unit staff will compare the returned study drugs with the dosage information reported by the subject and the prescribed dosage to monitor compliance. During each study visit, the number of returned study drugs and the dosing information reported by the subject will be reconciled with the subject, and compared with the prescribed dosing regimen to monitor compliance. Any deviations from compliance will be recorded in the source documents and the drug inventory records. Any deviations from compliance should be documented, along with an explanation for the deviation.

Calculation of dosage compliance:

% Compliance = (Number of tablets taken / Number of tablets expected to be taken) × 100.

If the percentage compliance calculated by the above formula is less than 80% or greater than 120%, the subject can be considered non-compliant with the prescribed dosage. If the subject's compliance falls outside the above range, it should be recorded as a protocol deviation. The study drug eCRF should reflect the verified dosage information provided by the subject.

6 Concomitant medication

6.1 Prohibited medications

During the treatment period, all other antitumor drugs and supportive medications related to cancer treatment, including traditional Chinese medicine for anti-tumor purposes and immunotherapeutic agents, should be discontinued, except for those specified in this protocol.

6.2 Medications to be used with caution

If subjects experience any adverse reactions, close monitoring should be conducted. Active symptomatic treatment should be provided if necessary, and the medications used should be documented and explained in the CRF. The following medications should be used with caution during the study period:

1) Drugs and foods that interfere with liver P450 enzymes:

- CYP3A4 inducers (dexamethasone, phenytoin, carbamazepine, rifampicin, rifabutin, rifapentine) and inhibitors (ketoconazole, itraconazole, erythromycin, clarithromycin, atazanavir, indinavir, nefazodone, nelfinavir,

ritonavir and saquinavir);

- CYP3A4 substrates (simvastatin, pimedium);
- Other drugs metabolized by CYP3A4 (e.g., benzodiazepines, dihydropyridines, calcium antagonists, HMG-COa reductase inhibitors);
- CYP2C9 substrates (diclofenac, phenytoin, piroxicam, S-warfarin, and tolbutamide) and CYP2C19 substrates (diazepam, imipramine, lansoprazole, and S-mephenytoin);

2) Drugs that prolong the QT interval: including antibiotics, antiarrhythmics, antipsychotics, antifungals, antimalarials and antidepressants (such as clarithromycin, quinidine, risperidone, fluconazole, mefloquine, amitriptyline, azithromycin, sotalol, fluphenazine, ketoconazole, chloroquine, imipramine, erythromycin, amiodarone, droperidol, clomipramine, Roxithromycin, disopyramide, haloperidol, dutiapine, metronidazole, procainamide, thioridazineDoxepin, moxifloxacin, pimozide, olanzapine, and clozapine.

6.3 Permitted Concomitant Medications and Treatments

Patients can receive optimal supportive care, and any clinical comorbidities and various AEs that arise should be actively treated. All concomitant medications must be strictly recorded in the CRF according to GCP regulations. Concomitant medications/treatments should be recorded from 4 weeks before the start of the study treatment until the end of the safety follow-up.

7 Study procedures

Before starting the study, patients must read and sign the informed consent form currently approved by the Ethics Committee (EC). All study procedures must be conducted within the time window specified in the study schedule.

7.1 Screening Period

After signing the informed consent form, subjects enter the screening period. Unless otherwise stated, the following screening steps must be completed within 14 days of the screening period:

- Demographic: initials, gender, ethnicity, marital status, date of birth, height, weight, and calculation of body surface area based on these;
- Medical history: past medical history and treatment history (clinical/pathological diagnosis, time of diagnosis, clinical/pathological stage, HER2/ER/PR expression status); whether surgery was performed; whether neoadjuvant therapy was administered; whether adjuvant therapy was given; whether radiotherapy was conducted; timing of progression; basis for diagnostic progression; whether late-stage systemic

treatment was received; names of drugs used in adjuvant and late-stage systemic treatments, methods of administration, duration of medication, efficacy; whether other treatment methods were used (such as bisphosphonate therapy); smoking and alcohol consumption history (frequency, amount, duration); drug allergy history (drug name, allergic symptoms); previous diseases or concurrent diseases/symptoms (disease/symptom name, names of concomitant medications, dosage, methods of administration, outcomes); bowel habits (frequency);

- ECOG score;
- Vital signs: body temperature, blood pressure, respiratory rate, and pulse;
- Physical examination: general condition, skin and mucous membranes, lymph nodes, head and neck, chest, abdomen, musculoskeletal, neurological reflexes, respiratory system, cardiovascular system, genitourinary system, and mental status, etc.;
- Blood routine;
- Virology Test;
- Thyroid function;
- Urine routine;
- Stool routine;
- Coagulation function;
- Blood biochemistry;
- Blood immune cell examination (the decision to conduct this examination is made by the physician based on specific conditions);
- Infectious disease screening;
- Pregnancy test;
- 12-lead ECG:
- Echocardiography: Reports within 28 days prior to enrollment can be used (including qualified cardiac ultrasounds completed before signing the ICF);
- Imaging Evaluation: At least includes chest CT, ultrasound (breast, neck, axillary, supraclavicular lymph nodes, abdomen, and gynecological), cranial MRI, bone scan, etc. Reports within 28 days prior to enrollment can be used (including qualified tumor imaging examinations completed before signing the ICF),

with bone scan results referring to those from the past year;

- Concomitant medication/treatment: should be recorded from within 28 days prior to enrollment;
- Adverse event follow-up: should be recorded from the day the subject signs the informed consent form until 28 days after the last medication.

7.2 Trial period

The following examinations should be completed within the time window specified in the trial protocol. If there are statutory holidays, they can be conducted earlier, and the reason for the window deviation should be recorded in the CRF. Investigators may add test items or increase the frequency of visits as appropriate.

- ECOG score: Assessed once at each visit. Investigators decided on tests based on actual conditions or clinical needs;
- Vital signs (including body temperature, blood pressure, respiratory rate, and pulse): check once at each visit. Investigators decided on tests based on actual conditions or clinical needs. Perform at the end of treatment or withdrawal (if not conducted within the previous 7 days);
- Physical examination: includes general condition, skin and mucous membranes, lymph nodes, head and neck, chest, abdomen, musculoskeletal, neurological reflexes, respiratory system, cardiovascular system, genitourinary system, and mental status. Check during the screening and trial periods (every 2 cycles ± 7 days); Perform at the end of treatment or withdrawal (if not conducted within the previous 7 days);
- Blood biochemistry: including Glucose, TP, A/G, ALT, AST, ALP, γ-GT, ALB, TBIL, DBIL, IBIL, TG, CHOL, UA, BUN, Cr, K+, Na+, CL-, Ca2+; To be checked once during the screening period and one day before or on the day of each medication visit. Additionally, during the first two treatment cycles, it is recommended to review weekly after the end of medication, with an interval of at least 5 days. Perform at the end of treatment or withdrawal (if not conducted within the previous 7 days). If necessary, myocardial enzyme spectrum tests can be conducted as determined by the researcher based on the subject's condition; this decision includes whether to accept test results from other hospitals;
- Blood Routine: includes of WBC, ANC, LC, RBC, Hb, and PLT; conducted once during the screening period and one day before or on the day of each medication visit.; Additionally, during the first two treatment cycles, it is recommended to review weekly after the end of medication, with at least a 3-day interval; Perform at the end of treatment or withdrawal (if not conducted within the previous 7 days). (the researcher determines whether to accept test results from other hospitals);
- Thyroid function: including serum thyroid-stimulating hormone (TSH), free triiodothyronine (FT3), and free thyroxine (FT4). If FT3 and FT4 are unavailable, T3 and T4 may be used as substitutes. During the screening period and trial period, conduct examinations every 2 cycles ± 7 days; Perform at the end of treatment or withdrawal (if not conducted within the previous 7 days);
- Urine routine: includes urine protein, if urine protein++ or higher, the 24-hour urine protein quantification should be assessed; Perform one assessment during the screening phase. Throughout the trial period, the investigator will decide on the necessity of further assessments based on the specific circumstances. Perform at the end of

treatment or withdrawal (if not conducted within the previous 7 days);

- Stool routine: includes stool characteristics and fecal occult blood. Perform one assessment during the screening phase. Throughout the trial period, the investigator will decide on the necessity of further assessments based on the specific circumstances. Perform at the end of treatment or withdrawal (if not conducted within the previous 7 days).;
- Coagulation function: including PT, INR, APTT, and TT. Examination will be performed during the screening period and at the end of treatment or withdrawal; Throughout the trial period, the investigator will decide on the necessity of further assessments based on the specific circumstances;
- Blood immune cell examination: including absolute lymphocyte count, absolute B lymphocyte count, B lymphocyte CD19 ratio, absolute T lymphocyte count, T lymphocyte CD3 ratio, absolute Th lymphocyte count, Th lymphocyte CD4 ratio, absolute Ts lymphocyte count, Ts lymphocyte CD8 ratio, CD4/CD8 ratio, absolute natural killer cell count, natural killer cell CD56+CD16 ratio, etc. The investigator will decide on the necessity of further assessments based on the specific circumstances;
- Pregnancy test: female subjects of childbearing age must undergo blood HCG testing during the screening period to exclude pregnancy. Test will be conducted during the screening period and at the end of treatment or withdrawal. The investigator will decide on the necessity of further assessments based on the specific circumstances during trial period;
- 12-lead ECG: pay attention to QT, QTc, and P-R intervals. Tests conducted before or on the day of each visit during the screening period and before medication. Perform at the end of treatment or withdrawal (if not conducted within the previous 7 days). During trial period, if the QTc interval increases by >30 msec from baseline, or QTc interval absolute value of ≥470 msec, two additional ECG examinations are required (at least 10 minutes apart). (the researcher determines whether to accept test results from other hospitals);
- Echocardiography: Conducted during the screening period and every 12 weeks ± 7 days during the trial period. If symptoms such as chest pain or palpitations occur;
- Imaging evaluation: the timing of tumor imaging examinations during the trial period is determined from the start of the study treatment, without considering the time paused for administration due to toxicity reactions. The trial period's examination items are decided by the clinical physician based on the actual situation. Imaging assessments will be conducted every 2 cycles ± 7 days. Confirmation of initial intracranial/extracranial partial response (PR) or complete response (CR) must be performed at least 4 weeks after the initial assessment. Investigators may increase the frequency of imaging assessments based on clinical needs;
- Concomitant medication/treatment: record concomitant medication during the trial at any time;
- Adverse events: observe and record adverse events during the study at any time;
- Drug recovery: collect the remaining drugs from the trial.

7.3 End of study treatment/withdrawal

Treatment will continue until disease progression, unacceptable toxicity, withdrawal of informed consent, or discontinuation of the study as determined by investigators. Disease progression is defined as intracranial lesion progression (assessed according to RANO-BM criteria) and/or extracranial lesion progression (assessed according to

RECIST criteria).

After the termination of treatment, the physician will provide subsequent treatment recommendations based on the patient's condition.

At the end of study treatment or withdrawal from the study, if the patient has not undergone assessments within 7 days prior to the end of the study (except for echocardiography and tumor imaging assessments), the following tests should be conducted:

- ECOG score;
- Vital signs;
- Physical examination;
- Blood routine: if not conducted within the previous 7 days;
- Thyroid function: if not conducted within the previous 7 days;
- Urine routine: if not conducted within the previous 7 days;
- Stool routine: if not conducted within the previous 7 days;
- Blood biochemistry: if not conducted within the previous 7 days;
- Pregnancy test;
- 12-lead ECG: if not conducted within the previous 7 days;
- Echocardiography: if not conducted within the prior 4 weeks;
- Imaging evaluation: if not conducted within the prior 8 weeks;
- Concomitant medication/treatment: record in real-time;
- Adverse events: record in real-time.
- Drug recovery: collect the remaining drugs from the trial.

7.4 Follow-up period

Subjects enter the follow-up period after withdrawal from the study, with the following requirements to be met until the completion of survival follow-up:

- Safety follow-up: adverse events that have not recovered after discontinuing the dosing regimen during the trial should be tracked and a final evaluation should be conducted. All subjects should undergo safety follow-up until 28 days after the last dose. Adverse events, concomitant medications/treatments, and unplanned tests or examinations during this period should be recorded in detail. Researchers should follow up on each adverse event until the event resolves to baseline level or better, the event is assessed as stable by the researcher, the subject is lost to follow-up, or the subject withdraws consent. Every effort must be made to follow up on all serious adverse events related to the investigational drug or caused by trial procedures until final results are available.
- Efficacy follow-up: all subjects are required to undergo efficacy follow-up. From the start of the follow-up period, they will be contacted every 12 weeks (±7 days) until the PFS data collection is completed or the subject is lost to follow-up, whichever occurs first. The details of each follow-up must be recorded and entered into the CRF.
- Survival follow-up: Subjects who have completed both safety and efficacy follow-ups (based on the later completion date) are required to undergo survival follow-up. Survival follow-up will be conducted every 12

weeks (±7 days) until the subject dies, lost to follow-up, or the end of OS data collection is reached, whichever occurs first. The details must be recorded and entered into the CRF.

8 Efficacy Evaluation

The primary endpoint of the study is to explore the intracranial objective response rate (CNS-ORR), according to the RANO-BM criteria, detailed in Appendix 5.

The secondary endpoints are to explore the intracranial clinical benefit rate (CNS-CBR: CR + PR + SD ≥ 24 weeks); progression-free survival (PFS)， including overall PFS (assessed by RANO-BM criteria and RECIST 1.1 criteria) and CNS-PFS (assessed by RANO-BM criteria); overall survival (OS); and the site of first progression.

The imaging evaluation of extracranial lesions is based on the the RECIST 1.1 criteria.

9 Safety Evaluation

The safety of the study drug will be evaluated through the recording of adverse events (including serious adverse events), laboratory tests, vital signs, physical examinations, ECOG scores, echocardiography, and electrocardiograms during the trial period. Subjects will be closely monitored for symptoms and signs after medication. Adverse events/reactions will be promptly and effectively managed to ensure the safety and interests of the subjects. After the timely and effective management of drug-related adverse events/reactions, their type, symptoms, occurrence time, severity (or grade), symptomatic treatment methods, and outcomes should be recorded. Subsequently, these adverse events should be analyzed, assessed, and statistically evaluated to provide a basis for the continuation of the trial.

9.1 Physical Examination and Vital Signs

The physical examination is conducted by the research physician and includes the following: general condition, skin and mucous membranes, lymph nodes, head and neck, chest, abdomen, musculoskeletal system, neurological reflexes, respiratory system, cardiovascular system, genitourinary system, and mental state. Weight is measured at each physical examination, while height is measured only once during the screening period.

Vital signs include the following: body temperature, blood pressure, respiratory rate, and pulse.

The ECOG score is evaluated by the research physician according to the content in Appendix 2: 'Performance Status Scoring Criteria (ECOG).'

9.2 Laboratory Examinations

Laboratory examination samples will be collected at the time points specified in the 'Flowchart'. As shown in Table 8, the following laboratory indicators will be sampled and tested by the research unit. For the safety of the subjects, unscheduled clinical laboratory tests may be performed at any time.

Table 8 Requirements for Laboratory Examinations

Blood Routine	Blood Biochemistry		Urine Routine [a]
White blood cell<br>Absolute neutrophil count<br>Lymphocyte count<br>Red blood cell<br>Hemoglobin<br>Blood platelet	Total bilirubin<br>Direct bilirubin<br>Indirect bilirubin<br>ALT<br>AST<br>Alkaline phosphatase<br>r-GT<br>Total protein<br>Albumin	Blood urea pitrogen<br>Creatinine<br>Uric acid<br>Blood glucose<br>Triglyceride<br>Cholesterol<br>Potassium<br>Sodium<br>Chlorine<br>Calcium	Urine protein<br>Urine glucose<br>Urine occult blood
Stool Routine	Coagulation Function	Virology Test	Others
Stool Characteristics<br>Stool Occult Blood	Prothrombin time<br>International normalized ratio<br>Activated partial thromboplastin time<br>Thrombin time	Hepatitis B Five Items<br>HCV Antibody<br>HBV-DNA<br>HCV-RNA<br>HIV Antibody	Pregnancy test [b]<br>Blood immune cell examination [c]<br>Thyroid function test

Note:

a. If the semi-quantitative method shows protein ≥2+ (e.g., urine dipstick), a 24-hour urine protein quantification test should be conducted.

b. Women of childbearing age need to undergo blood HCG testing during the screening period to exclude pregnancy, while urine HCG testing can be performed at other time points.

c. Blood immune cell examination should be determined by the clinical physician based on the detection conditions of each center and the needs of the patient's condition.

9.3 Electrocardiogram

The 12-lead electrocardiogram will be the responsibility of a qualified physician at the time points specified in the "Flowchart." All electrocardiogram examinations must be conducted after the subjects have rested in a quiet, supine position for at least 10 minutes. The content must include at least: heart rate, QT interval, QTc interval, and P-R interval.

To assess the safety of the subjects, the physician will compare the ECG results with the baseline measurements. If the QTc interval increases by more than 30 msec compared to the baseline, or if any single scheduled ECG measurement shows an absolute QTc interval value of ≥470 msec, two additional ECG tests should be conducted, with a recommended interval of at least 10 minutes, to ensure the accuracy of the initial measurement and to rule out abnormal ECG results due to incorrect lead placement. If the machine-read QTc value is prolonged, as described above, and a qualified physician determines that the QTc value is within an acceptable range, repeat measurements may not be required.

9.4 Echocardiography

Echocardiography will be performed by a qualified physician at the time points specified in the 'Flowchart.' During the administration of the investigational drug, the research physician will evaluate and monitor LVEF at the time points specified in the protocol. If the subject develops symptoms of heart failure or clinically significant decreases in LVEF, as determined by the researcher, treatment and monitoring should follow standard medical guidelines, and consultation with a cardiologist should be sought if necessary. If there are symptoms of severe heart failure that are clinically uncontrollable (NYHA Class III or IV) or significant decreases in LVEF (below the lower limit of normal or below 50%), administration of the investigational drug should be discontinued according to the 'Dose Adjustment' specified in Section 5.4, and treatment and monitoring should continue according to standard medical guidelines.

9.5 Collection and Reporting of Adverse Events (AE) and Serious Adverse Events (SAE)

9.5.1 Adverse Events

The collection of AE information begins when the subject signs the informed consent form and continues until the end of the safety follow-up period (28 days after the last dose of medication).

9.5.2 Definition of Adverse Events

Adverse Event (AE) refers to an unfavorable medical occurrence that appears after a subject in a clinical trial receives a drug, but it does not necessarily have a causal relationship with the treatment. AE can be any unfavorable and unintended symptoms, signs, abnormal laboratory findings, or diseases, and at least include the following situations:

1) Worsening of pre-existing (before entering the clinical trial) medical conditions/diseases (including worsening of symptoms, signs, or laboratory abnormalities);

2) Any newly occurring AE: Any newly occurring adverse medical condition (including symptoms, signs, newly diagnosed diseases);

3) Abnormal laboratory test values or results of clinical significance.

Researchers should meticulously document any AE occurring in subjects, including: the name of the AE and a description of all related symptoms, time of occurrence, severity, correlation with the investigational drug, duration, measures taken regarding study medication, and final outcome and disposition.

9.5.3 Criteria for Severity of Adverse Events

The adverse events will be graded in accordance with the NCI-CTCAE Version 5.0

If an adverse event not listed in the NCI-CTCAE version 5.0, the following criteria can be referred to:

Grade 1: Mild; asymptomatic or mild symptoms; clinical or diagnostic observations only; intervention not indicated.

Grade 2: Moderate; minimal, local or noninvasive intervention indicated; limiting age-appropriate instrumental ADL.

Grade 3: Severe or of significant medical importance but not immediately life-threatening; leads to hospitalization or prolongation of hospitalization; causes disability; limits self-care activities of daily living (Self-care ADL). Self-care ADL refers to activities such as bathing, dressing, undressing, eating, toileting, and taking medication, with the subject not being bedridden.

Grade 4: Life-threatening consequences; requires urgent intervention.

Grade 5: Death related to AE.

9.5.4 Criteria for Assessing the Relationship between Adverse Events and the Investigational Drug

The collection of AEs begins upon signing the informed consent form and continues until the end of the safety follow-up period. Regardless of whether the event is related to the investigational drug, whether the subject is assigned to the investigational drug group, or whether the drug is used, it must be collected and recorded. Any discomfort reported by the subjects during the treatment period or any abnormal changes in objective laboratory test indicators should be truthfully recorded. At the same time, the severity, duration, management measures, and outcomes of the AE should be noted. The researcher should comprehensively determine the relationship between the AE and the investigational drug, such as whether the occurrence of the AE has a reasonable temporal sequence with the medication, the characteristics of the investigational drug, its toxicological and pharmacological effects, whether the subject is using other concomitant medications, the subject's underlying diseases, medical history, family history, as well as provocation and re-provocation reactions, etc. An assessment should be made of the possible association between adverse events and the investigational drug according to the five-tier classification: "definitely related, possibly related, possibly unrelated, definitely unrelated, and indeterminable." The assessment criteria are shown in Table 9:

Table 9 Criteria for Assessing the Relationship between Adverse Events and the Investigational Drug

Grading	Criteria
Definitely Related	The occurrence of the event follows a reasonable time sequence after medication, the event matches the known reaction type of the suspected drug; improvement occurs after discontinuation, and the event reappears upon re-administration.
Possibly Related	The occurrence of the event follows a reasonable time sequence after medication, but the event does not match the known reaction type of the suspected drug; the patient's clinical status or other treatments could also potentially cause the event.

Possibly Unrelated	The occurrence of the event does not follow a reasonable time sequence after medication, nor does it match the known reaction type of the suspected drug, and the patient's clinical status or other treatments could potentially cause the event.
Definitely unrelated	The event does not occur in a reasonable chronological order following medication, the event does not align with the known reaction types of the suspected drug, the patient's clinical condition or other treatments may have caused the event, the event disappears upon disease improvement or cessation of other treatments, and the event reappears with the repeated use of other treatments.
indeterminable	The occurrence of the event has no clear temporal relationship with the medication, it resembles the known reaction types of the drug, and other concurrently used medications may also cause the corresponding event.

9.5.5 Serious adverse events

A serious adverse event (SAE) refers to a medical occurrence during a clinical trial that requires hospitalization or prolongs hospitalization, results in disability, affects the ability to work, is life-threatening or results in death, or causes congenital anomalies. Medical events that meet one or more of the following criteria are considered SAEs:

- Events leading to death;
- Life-threatening events (defined as events where the subject is at immediate risk of death at the time of the event);
- Events leading to hospitalization or prolongation of hospitalization;
- Events causing permanent or severe disability/incapacity/impact on work ability;
- Congenital anomalies or birth defects;
- Other important medical events (defined as events that jeopardize the subject or require intervention to prevent any of the above outcomes).

9.5.6 Hospitalization

Adverse events in clinical trial that lead to hospitalization (for less than 24 hours) or prolongation of hospitalization should be considered SAEs. The following hospitalization situations, however, do not constitute SAEs:

- Rehabilitation facilities
- Sanatoriums
- Routine emergency room admissions
- Same-day surgeries (such as outpatient/same-day/non-bedridden surgeries)
- Hospitalization or an extension of a hospital stay that is not attributable to the exacerbation of an adverse event is not classified as a serious adverse event (SAE). For example:
    - ✧ Hospitalized due to pre-existing conditions, with no new adverse events occurring and no worsening of the pre-existing conditions (e.g., persistent laboratory abnormalities from before the trial to the present).
    - ✧ Hospitalization for administrative reasons (e.g., annual routine check-ups).
    - ✧ Hospitalization as required by the trial protocol during the clinical trial (e.g., procedures specified by the trial protocol).

- ✧ Elective hospitalization unrelated to the worsening of adverse events (e.g., elective surgery).
- ✧ Scheduled treatments or surgeries should be recorded in the trial protocol and/or in the subject's personal baseline data.
- ✧ Admission solely for the use of blood products.

Diagnostic or therapeutic invasive (e.g., surgical) and non-invasive procedures should not be reported as adverse events. However, if the disease condition leading to the procedure meets the definition of an adverse event, it should be reported. For instance, acute appendicitis occurring during the adverse event reporting period should be reported as an adverse event, while the appendectomy performed as a result should be recorded as the treatment method for that adverse event.

9.5.7 Disease progression and death

Disease progression is defined as the worsening of the subject's condition caused by the indication under study, including radiographic progression and the progression of clinical symptoms and signs. New metastatic lesions of the primary tumor or the progression of existing metastatic lesions are considered disease progression. Events caused by symptoms and signs of disease progression that are life-threatening, require hospitalization or prolongation of hospital stay, or result in permanent or severe disability, functional impairment, impact on work ability, congenital anomalies, or birth defects are not reported as Serious Adverse Events (SAEs). If there is any uncertainty about whether the SAE is caused by disease progression, it should be reported as an SAE.

In the study population of this trial, "disease progression" is an expected occurrence and should not be reported under the term of Adverse Event (AE). When disease progression occurs, the event used to confirm disease progression should be reported as an AE. For instance, if subject experiences a seizure that is determined to be related to brain metastasis, the AE term should be recorded as "seizure" rather than "disease progression" or "brain metastasis."

If subject dies during the trial period, regardless of whether they have received new anti-tumor treatment, it must be reported as a Serious Adverse Event (SAE) (refer to Table 11 for the principles of AE/SAE collection and follow-up periods). Deaths that are assessed by the investigator as potentially caused by symptoms and signs of disease progression should be recorded in the electronic Case Report Form (eCRF) and reported as an SAE. The term "death" should not be used as an AE or SAE term; instead, the event that caused or led to death should be recorded as an AE or SAE. If the cause of death is unknown and cannot be determined at the time of reporting, the AE or SAE term should be recorded as "death due to unknown causes."

9.5.8 Additional Antitumor Treatment

The documentation of Serious Adverse Events (SAEs) begins when the subject signs the informed consent form and continues until the end of the safety follow-up period (28 days after the last administration of the investigational drug). If a subject starts other antitumor treatment before the end of the safety follow-up period, for non-fatal SAEs, the reporting period ends when the new antitumor treatment begins, unless there is suspicion of a relation to the investigational drug. If death occurs within the safety follow-up period, it must be reported as an SAE regardless of whether the subject has received other treatments.

9.5.9 SAE Reporting System

The collection period for SAEs should begin when the subject signs the informed consent form and continue until the end of the safety follow-up period. In the event of an SAE, whether it is an initial or follow-up report, the researcher must immediately complete the Serious Adverse Event Report Form, sign and date it. The investigator must notify the sponsor within 24 hours of becoming aware of the SAE, report to the Hengrui Drug Safety Department, and promptly report to the relevant authorities as required by regulations.

The email address for the Hengrui Drug Safety Department to receive SAE reports is: hengrui_drug_safety@hrglobe.cn.

SAEs occurring after the safety follow-up period should be collected if they are suspected to be related to the investigational drug. SAEs should be recorded in detail, including symptoms, severity, relevance to the trial medication, time of occurrence, time of management, measures taken, follow-up timing and method, and outcome. If the investigator believes that a certain SAE is unrelated to the trial medication and potentially related to study conditions (e.g., discontinuation of previous treatment, or comorbidities during the trial), this relationship should be detailed in the narrative section of the SAE report form. If there is a change in the intensity of an ongoing SAE or its relationship with the investigational drug, a follow-up report should be submitted immediately. If the investigator believes that previously reported SAE information was incorrect, corrections, withdrawals, or downgrades can be made in the follow-up report and submitted in accordance with the SAE reporting procedures.

9.5.10 Abnormal Liver Function

If AST and/or ALT levels, along with total bilirubin levels, are abnormally elevated, and the following conditions (1), (2), and (3) are met without any other identifiable causes for the abnormality, such cases should always be reported as SIEs (Serious Infections). If they also meet the criteria for SAEs (Serious Adverse Events), they must be reported

according to the SAE procedure as well.

Table 10: Criteria for Evaluating Potential Drug-Induced Hepatotoxicity

Conditions	Criteria
(1) Abnormal ALT or AST	Normal at baseline: During treatment, ALT or AST ≥ 3×ULN;<br>Abnormal at baseline: During treatment, ALT or AST ≥ 2× baseline level and value ≥ 3× ULN; or value ≥ 8× ULN.
(2) Abnormal TBIL	Normal at baseline: During treatment, TBIL > 2×ULN;<br>Abnormal at baseline: During treatment, TBIL increase ≥ 1×ULN or its value > 3×ULN.
(3) No hemolysis and alkaline phosphatase < 2×ULN (or information not obtained)	

If a subject experiences abnormally elevated levels of AST and ALT concurrently with increased total bilirubin levels during the treatment period or the follow-up period, they should return to the research center for examination and assessment as soon as possible (ideally within 48 hours) after being informed of the abnormal results. This should include laboratory tests, a detailed medical history inquiry, and a physical evaluation, with consideration given to the possibility of liver tumors (primary or secondary).

In addition to repeating tests for AST and ALT, laboratory tests should also include albumin, creatine kinase, total bilirubin, direct and indirect bilirubin, γ-glutamyl transferase, prothrombin time (PT)/international normalized ratio (INR), and alkaline phosphatase. A detailed medical history should include: alcohol consumption history, acetaminophen use, soft drug use, use of various supplements, family medical history, occupational exposure, sexual behavior history, travel history, contact with jaundice patients, surgeries, blood transfusions, and history of liver or allergic diseases. Further examinations may also include testing for acute hepatitis A, B, C, and E, as well as liver imaging studies (such as bile duct imaging). If repeated tests still confirm the definition according to the above laboratory standards, and there is no other reason for liver enzyme abnormalities, it should be reported as an SAE.

9.5.11 Pregnancy

During the clinical study, if a female subject becomes pregnant, she will be withdrawn from the study. If a male subject's partner becomes pregnant during the clinical study, the male subject will continue to participate in the study. The investigator should complete the "Hengrui Clinical Study Pregnancy Report/Follow-up Form" and report it to the sponsor within 24 hours of learning about the pregnancy and promptly report to the ethics committee.

The investigator should follow up on the pregnancy outcome, continuing follow-up until one month after the mother's delivery, and report the results to the sponsor. If the pregnancy outcome is stillbirth, spontaneous abortion,

or fetal deformity, it is considered a Serious Adverse Event (SAE) and must be reported according to the SAE reporting timeline requirements.

If the subject experiences an SAE during the pregnancy, the "Serious Adverse Event Report Form" must also be completed, and the SAE reporting procedures must be followed for submission.

9.5.12 Follow-up of AE/SAE

All AEs/SAEs should be followed up until the end of the safety follow-up period, until they resolve or are reduced to baseline levels or to Grade≤1, reach a stable state, or are reasonably explained (such as loss to follow-up, death).

Investigators should inquire about any AEs/SAEs that have occurred since the last visit during each visit and provide follow-up information in a timely manner as required by the sponsor's queries. For the collection and follow-up period of AEs/SAEs that occur after the last administration of medication at the end of the study, please refer to Table 11 below:

Table 11 Principles for AE/SAE Collection and Follow-up Period

Category	Collection/Recording Requirements	Follow-up Requirements
Non-drug-related AE	Until the end of the safety follow-up period or the start of new antitumor therapy (whichever comes first)	Until the end of the safety follow-up period
Drug-related AE	Until the end of the safety follow-up period	Follow-up until disappearance, alleviation, return to baseline level, ≤ Grade 1, reaching a stable state, or obtaining a reasonable explanation (e.g., loss to follow-up, death)
Non-drug-related SAE	Until the end of the safety follow-up period or the start of new antitumor therapy (whichever comes first)	Until the end of the safety follow-up period
Drug-related SAE	Indefinite	Follow-up until disappearance, alleviation, return to baseline level, ≤ Grade 1, reaching a stable state, or obtaining a reasonable explanation (e.g., loss to follow-up, death)

AE: Adverse Events; SAE: Serious Adverse Events

10 Research Management

10.1 Ethical and Informed Consent

10.1.1 Ethical

This clinical trial must comply with the Declaration of Helsinki (2008 version), the Good Clinical Practice (GCP) guidelines issued by the NMPA (formerly known as CFDA), and relevant regulations. Prior to the commencement of

the trial, approval must be obtained from the ethics committee of the responsible organization to initiate this study. During the clinical research, any modifications to the trial protocol must be reported to and reviewed by the ethics committee. The researcher is responsible for regularly submitting interim trial reports in accordance with the requirements of the ethics committee. After the trial concludes, the researcher should inform the ethics committee that the trial has been completed.

10.1.2 Informed Consent

Before receiving the administration of this trial medication, subjects must provide informed consent to participate in order to protect their legal rights. The researcher is responsible for thoroughly and comprehensively explaining to the subjects or their designated representatives the purpose of the study, the effects of the drug, potential side effects, and possible risks. Subjects should be made aware of their rights, the risks they are undertaking, and the benefits. This conversation is a crucial part of the informed consent process. If the subject or their legal representative is illiterate, a witness should be present during the informed consent process. After the subject or their legal representative gives verbal consent, they should sign the informed consent form on the same day, with the witness also signing. The informed consent form should indicate the version number and the version date.

10.2 Management of Study Drugs

The management, distribution, and collection of clinical trial medications in this study will be handled by designated personnel. The researcher must ensure that all investigational drugs are used exclusively for the subjects participating in this clinical trial. The dosage and administration should adhere to the trial protocol, and any remaining drugs should be returned to the sponsor. Clinical trial medications must not be transferred to any non-participants of the trial.

Monitors are responsible for overseeing the supply, usage, storage, and disposal of remaining investigational drugs in the clinical trial.

10.3 Protocol Amendments

No one other than the principal investigator is allowed to make modifications to the protocol. Any necessary changes to the protocol must be made in the form of amendments. These amendments must be submitted to the ethics committee for approval or record after obtaining the principal investigator's signed consent, and the protocol must include detailed explanations of each modification.

10.4 Monitoring

The investigator appoints individuals with appropriate medical, pharmaceutical, or related professional

qualifications, and who have undergone necessary training, are familiar with Good Clinical Practice (GCP) and relevant regulations, to serve as monitors for this clinical trial. Their role is to oversee and report on the conduct of the trial, verify data, ensure the rights and welfare of the subjects are protected, and guarantee that trial records and reported data are accurate and complete. They ensure that the trial adheres to the approved protocol, GCP, and relevant regulations.

By monitoring adverse events (AEs) and serious adverse events (SAEs) during the clinical trial, they ensure that all AEs are recorded and reported accurately, reliably, and promptly.

10.5 Quality Control and Assurance

- The clinical research unit must be a drug clinical research base with the conditions for clinical research as determined by the NMPA.
- Researchers must be physicians trained in clinical trials and work under the guidance of senior professionals.
- The clinical wards must meet standardized requirements before the trial, ensuring that rescue equipment is complete.
- Medication should be administered by professional nursing staff who thoroughly understand the medication regimen to ensure subject compliance.
- Each research center must strictly adhere to the study protocol and accurately complete the CRF.
- Monitors should follow standard operating procedures to oversee the conduct of the clinical trial, ensuring that all data recording and reporting are accurate and complete, that all CRFs are correctly filled out, and consistent with original documents, thus ensuring the trial is conducted according to the clinical research protocol.
- In the event of an SAE, monitors must promptly inform all trial units, and if necessary, temporarily halt the study.
- The participating centers in the trial should accept inspections by drug regulatory authorities. It is particularly important for the researchers and their associated personnel to facilitate and allocate time for monitoring and inspections.

10.6 Data Management

10.6.1 Data Collection

This study uses a Case Report Form (CRF) for the collection of research data; personnel at the research institution must undergo systematic training.

10.6.2 Data Management and Quality Control

To ensure the authenticity and reliability of clinical trial data and to improve the quality of clinical data, clinical monitors will review the trial data in the clinical database for completeness, consistency, and accuracy according to

standard operating procedures during the trial process, and will guide research institution personnel in making necessary additions or corrections to problematic data. If clinical staff or data management personnel have questions about the data, they must raise concerns with the researchers or data entry personnel. The relevant personnel must respond to the concerns and correct or explain the problematic data. If necessary, multiple inquiries can be made until the data issues are resolved. Medical personnel and data management staff regularly conduct consistency checks on SAEs.

At the end of the trial project, data management personnel and medical staff will perform the final quality control on all data, summarize all protocol deviations and violations that occurred during the trial, and hold a data review meeting. Data analysis can only be conducted by statistical personnel after all data meet quality requirements.

10.6.3 Review of Data and Monitoring of Research Institutions

Before the trial starts, the principal investigator must introduce the trial protocol and the CRF. During the trial, the data monitoring leader must regularly check the completeness of patient records and the accuracy of the content filled in the CRF, compliance with the trial protocol and Good Clinical Practice (GCP) guidelines, and the progress of enrollment. They must ensure that the investigational drug is stored, distributed, and accounted for according to regulations. During this period, key researchers must be available to assist the data monitoring personnel.

Researchers must retain the original files of each patient participating in the trial, including research medical records and visit records (inpatient or outpatient medical records), which contain demographic indicators, medical information, laboratory data, electrocardiogram results, and any other examination or evaluation results. All information on the CRF must originate from the original documents in the patient's records. The researcher must also retain the signed informed consent form from the patient. The researcher must ensure that all relevant original documents are available for monitoring to confirm their consistency with the CRF content. Monitoring standards require 100% monitoring of obtained informed consent forms, compliance with inclusion/exclusion criteria, SAE records, and all data needed for evaluating primary and safety endpoints. According to the monitoring plan specified by the trial, additional checks on the consistency between the original data and the CRF should be conducted. Any information related to patient identity in the original documents will not be disclosed.

10.7 Protocol Violations

All requirements specified in the study protocol must be strictly adhered to. Any intentional or unintentional deviation or violation of the trial protocol and Good Clinical Practice (GCP) principles can be classified as a protocol

deviation or violation. During the monitoring process, if a protocol deviation is identified, the researcher or monitor should complete a protocol violation record, detailing the time of discovery, the time and process of the event, the cause, and the corresponding corrective measures. This record should be signed by the researcher and reported to the ethics committee.

10.8 Data Retention

To ensure evaluation and supervision of the clinical research by the National Medical Products Administration (NMPA) and the principal investigator, the researcher should agree to retain all study materials. These include the original records of the subjects' hospitalizations, informed consent forms, case report forms (CRFs), detailed records of drug distribution, etc. The study materials should be kept by the research institution for five years after the clinical trial ends. All materials of this clinical study are jointly owned by the principal investigator and their affiliated institution, Fudan University Shanghai Cancer Center, and the project supporter, Jiangsu Hengrui Pharmaceuticals Co., Ltd. Except as required by the NMPA, no one may provide them to a third party in any form without the joint written consent of all owners.

10.9 Publication

All articles and reports related to the trial must be approved by the principal research institution before they can be publicly published.

11 Statistic Analysis

11.1 Sample Size Calculation

The sample size is estimated based on the primary endpoint CNS-ORR. Since the limited data reported at the time of ABC study design, referring to the historical data on platinum-based regimen in solid tumor BMs and iniparib-based regimen in TNBC BMs, the null hypothesis for CNS-ORR was set at 14%[1,2], the alternative hypothesis for CNS-ORR was set at 34%. With a one-sided α of 0.05 and a power of 80%, 13 subjects would be enrolled at the first stage, the trial should be discontinued if 2 or fewer respond to the treatment. If the trial continues to the second stage and additional 18 patients should be enrolled. Overall, the null hypothesis was rejected and further investigation was favored only if more than 7 responders are obtained. Accounting for a 10% dropout rate, a total of 35 subjects were required to be enrolled.

11.2 Statistical Analysis Plan

11.2.1 Statistical Analysis Datasets

The analysis population for this study includes the Full Analysis Set (FAS), the Per Protocol Set (PPS), and the Safety Set (SS).

Full Analysis Set (FAS): This is the analysis set determined according to the intention-to-treat principle. It includes all enrolled subjects who have at least one medication record. The FAS is the primary analysis set for the efficacy analysis in this study.

Per Protocol Set (PPS): This is a subset of the full analysis set. It includes all enrolled subjects who have at least one medication record and have not experienced major protocol violations deemed to affect the efficacy analysis.

The efficacy analysis of the investigational drug is conducted on both the FAS and the PPS.

Safety Set (SS): This includes all enrolled subjects who have at least one medication record.

The safety analysis of the investigational drug is conducted on the Safety Set.

11.2.2 Statistical Analysis Methods

11.2.2.1 Basic Methods

In this study, unless otherwise specified, data will be summarized using descriptive statistics according to the following general principles.

Measurement data will be summarized using mean, standard deviation, median, maximum, and minimum values. Count data will be summarized using frequency and percentage, with a 95% confidence interval for the percentage provided when necessary. Time-to-event data will be analyzed using Kaplan-Meier estimates to calculate survival rates and plot survival curves.

11.2.2.2 Efficacy Endpoint Analysis

The primary endpoint is CNS ORR, and the analysis will be conducted on both the Full Analysis Set (FAS) and Per Protocol Set (PPS), with the FAS serving as the primary analysis set. A descriptive analysis will be performed for intracranial clinical benefit rate, progression-free survival, overall survival, and the site of first progression.

11.2.2.3 Safety Analysis

AEs will be coded using MedDRA and graded according to the NCI-CTCAE 5.0 grading system. TEAEs are defined as any adverse events that newly occur or worsen compared to baseline (prior to study treatment) after the initiation of the investigational drug.

Descriptive analysis will primarily be used to summarize data on AEs, serious adverse events (SAEs), grade ≥3 AEs, grade ≥3 SAEs, drug-related AEs, drug-related SAEs, AEs leading to dose adjustments, and AEs leading to treatment termination.

Describe the situation where laboratory test results are normal prior to the trial but abnormal after treatment, and the relationship of such abnormal changes with the investigational drug.

Summarize the difference in vital signs from baseline according to group and visit.

Summarize the baseline and post-baseline highest scores of ECOG-PS according to group.

Summarize the baseline and worst clinical abnormality grades post-baseline for ECG and echocardiogram according to group.

Safety analysis includes but is not limited to the above analyses.

11.2.3 Statistical software

Analysis using SAS version 9.4 or higher.

12 Subject Dropout

All subjects who have signed the informed consent form and have been screened and qualified to enter the trial have the right to withdraw from the clinical trial at any time. Subjects who withdraw at any time or for any reason without completing one cycle of the clinical trial and are unable to undergo safety and efficacy evaluations are considered dropouts (those who withdraw due to disease progression with clear medical evidence are not considered dropouts). When a subject withdraws, the researcher must record the reason for withdrawal in the CRF, complete any assessments that can be completed, and carefully document the final visit record in the CRF. For those who withdraw due to adverse reactions and are finally judged to be related to the investigational drug upon follow-up, this should be recorded in the CRF, and the researcher should be notified. Subjects who were only screened and did not obtain a drug number before withdrawing from the study are not considered dropout cases. If a subject completes a full course of treatment with detailed records, they should be included in the statistical analysis for the safety evaluation. Subjects who withdraw from the study cannot re-enter, and their numbers cannot be reused.

13 Forms of Research Publication

Publish an SCI paper and present an abstract at an international oncology conference.

14 References

1. Viñolas N, Graus F, Mellado B, et al: Phase II trial of cisplatinum and etoposide in brain metastases of solid tumors. J Neurooncol 35:145-8, 1997
2. Anders C, Deal AM, Abramson V, et al: TBCRC 018: phase II study of iniparib in combination with irinotecan to treat progressive triple negative breast cancer brain metastases. Breast Cancer Res Treat 146:557-66, 2014
3. Bertolini F, Spallanzani A, Fontana A, et al: Brain metastases: an overview. CNS Oncol 4:37-46, 2015
4. Tsukada Y, Fouad A, Pickren JW, et al: Central nervous system metastasis from breast carcinoma. Autopsy study. Cancer 52:2349-54, 1983
5. Patanaphan V, Salazar OM, Risco R: Breast cancer: metastatic patterns and their prognosis. South Med J 81:1109-12, 1988
6. Chien AJ, Rugo HS: Emerging treatment options for the management of brain metastases in patients with HER2-positive metastatic breast cancer. Breast Cancer Res Treat 137:1-12, 2013
7. Franciosi V, Cocconi G, Michiara M, et al: Front-line chemotherapy with cisplatin and etoposide for patients with brain metastases from breast carcinoma, nonsmall cell lung carcinoma, or malignant melanoma: a prospective study. Cancer 85:1599-605, 1999
8. Hu XC, Zhang J, Xu BH, et al: Cisplatin plus gemcitabine versus paclitaxel plus gemcitabine as first-line therapy for metastatic triple-negative breast cancer (CBCSG006): a randomised, open-label, multicentre, phase 3 trial. Lancet Oncol 16:436-46, 2015
9. Kolarić K, Roth A, Jelicic I, et al: Phase II clinical trial of cis dichlorodiammine platinum (Cis DDP) in metastatic brain tumors. J Cancer Res Clin Oncol 104:287-93, 1982
10. Groen HJ, Smit EF, Haaxma-Reiche H, et al: Carboplatin as second line treatment for recurrent or progressive brain metastases from small cell lung cancer. Eur J Cancer 29a:1696-9, 1993
11. Brufsky AM, Hurvitz S, Perez E, et al: RIBBON-2: a randomized, double-blind, placebo-controlled, phase III trial evaluating the efficacy and safety of bevacizumab in combination with chemotherapy for second-line treatment of human epidermal growth factor receptor 2-negative metastatic breast cancer. J Clin Oncol 29:4286-93, 2011
12. Zhuang H, Shi S, Yuan Z, et al: Bevacizumab treatment for radiation brain necrosis: mechanism, efficacy and issues. Mol Cancer 18:21, 2019
13. Lin NU GR, Younger J et al: Phase II trial of carboplatin (C) and bevacizumab (BEV) in patients (Pts) with breast cancer brain metastases (BCBM). J Clin Oncol 31, Abstract 513 2013
14. Schmid P, Chui SY, Emens LA: Atezolizumab and Nab-Paclitaxel in Advanced Triple-Negative Breast Cancer. Reply. N Engl J Med 380:987-988, 2019
15. Lauko A, Thapa B, Venur VA, et al: Management of Brain Metastases in the New Era of Checkpoint Inhibition. Curr Neurol Neurosci Rep 18:70, 2018
16. Gadgeel SM, Lukas RV, Goldschmidt J, et al: Atezolizumab in patients with advanced non-small cell lung cancer and history of asymptomatic, treated brain metastases: Exploratory analyses of the phase III OAK study. Lung Cancer 128:105-112, 2019
17. Lin NU, Lee EQ, Aoyama H, et al: Response assessment criteria for brain metastases: proposal from the RANO group. Lancet Oncol 16:e270-8, 2015

Appendix 1. Clinical staging criteria for breast cancer (8th edition AJCC breast cancer TNM staging)

Stage 0	TisN0M0
Stage IA	T1N0M0
Stage IB	T0N1miM0
	T1N1miM0
Stage IIA	T0N1M0
	T1N1M0
	T2N0M0
Stage IIB	T2N1M0
	T3N0M0
Stage IIIA	T0N2M0
	T1N2M0
	T2N2M0
	T3N1M0，T3N2M0
Stage IIIB	T4N0M0，T4N1M0，T4N2M0
Stage IIIC	Any T, N3M0
Stage IV	Any T, Any N, M1

Appendix 2. Physical Status Scoring Criteria (ECOG)

(Eastern Cooperative Oncology Group)

Scoring	Activity Level
0	Perfectly normal, able to carry out all normal activities without restriction
1	Incapable of strenuous physical activity but ambulatory and able to engage in light physical activity or office work
2	Can walk, can take care of oneself, but can not carry out any work, the time in bed during the day does not exceed 50 percent
3	They can barely take care of themselves and spend more than 50% of the day in bed or sitting in a chair.
4	Complete loss of mobility, severe inability to take care of oneself, and the need to lie in bed or use a wheelchair
5	Die

Appendix 3. Calculation of Creatinine Clearance Rate (Ccr)

Calculated using the Cockcroft-Gault formula:

Ccr (ml/min) (male) = (140 - age) × weight (kg) / [0.818 × serum creatinine (μmol/L)]

Ccr (ml/min) (female) = Ccr (male) × 0.85

Appendix 4. Adverse Reaction Evaluation Criteria (NCI-CTCAE Version 5.0, Partial Content)

	**Hematology/Bone Marrow Grading**				
Adverse Event	Grade 1	Grade 2	Grade 3	Grade 4	Grade 5
Anemia	Hb< LLN-100g/L	Hb < 100-80g/L	Hb < 80g/L	Life-threatening; urgent intervention	Death
Hemolysis	Laboratory evidence of hemolysis only	Hemolysis with ≥2g decrease in hemoglobin	Requires transfusion or medication (e.g., corticosteroids)	Life-threatening; urgent intervention	Death
Leukopenia	<LLN-3.0×10^9/L	<3.0-2.0×10^9/L	<2.0-1.0×10^9/L	<1.0×10^9/L	-
Lymphocytopenia	<LLN-0.8×10^9/L	<0.8-0.5×10^9/L	<0.5-0.2×10^9/L	<0.2×10^9/L	-
Neutropenia	<LLN-1.5×10^9/L	<1.5-1.0×10^9/L	<1.0-0.5×10^9/L	<0.5×10^9/L	-
Febrile Neutropenia	-	-	ANC<1000/mm^3 and a single temperature >38.3°C or body temperature ≥38°C lasting more than 1 hour	Life-threatening	Death
Thrombocytopenia	<LLN-75.0×10^9/L	<75.0-50.0×10^9/L	<50.0-25.0×10^9/L	<25.0×10^9/L	-
	**Hepatobiliary, Renal and urinary disorders**				
Adverse Event	Grade 1	Grade 2	Grade 3	Grade 4	Grade 5
Alkaline Phosphatase Increase	>ULN to 2.5xULN	>2.5xULN to 5.0xULN	>5.0xULN to 20.0xULN	>20.0xULN	-
Bilirubin	>ULN to 1.5xULN	>1.5xULN to 3.0xULN	>3.0xULN to 10.0xULN	>10.0xULN	-
GGT (Gamma-Glutamyl Transferase)	>ULN to 2.5xULN	>2.5xULN to 5.0xULN	>5.0xULN to 20.0xULN	>20.0xULN	-
Hypoalbuminemia	<LLN to 3 g/dL	<3 to 2 g/dL	<2 g/dL	Life-threatening; urgent intervention	Death
SGOT (AST) (Serum Aspartate Aminotransferase)	>ULN to 3.0xULN	>3.0xULN to 5.0xULN	>5.0xULN to 20.0xULN	>20.0xULN	-
SGPT (ALT) (Serum Alanine Aminotransferase)	>ULN to 3.0xULN	>3.0xULN to 5.0xULN	>5.0xULN to 20.0xULN	>20.0xULN	-
Liver - Other (For details, see below)	Asymptomatic or mild symptoms	Moderate symptoms	Severe symptoms	Life-threatening; urgent intervention	Death
Creatinine	>ULN to 1.5xULN	>1.5xULN to 3.0xULN	>3.0xULN to 6.0xULN	>6.0xULN	-
Proteinuria	1+: Protein in urine ≥ULN - ＜ 1.0g/24hrs	2+ and 3+: Protein in urine 1.0-- ＜ 3.5g/24hrs	4+: Protein in urine≥3.5g/24hrs	-	-

Appendix 5. RANO-BM Efficacy Evaluation Criteria[17]

Table 1 Summary of the response criteria for CNS metastases proposed by RANO-BM

	Complete response	Partial response	Stable disease	Progressive disease
Target lesions	None	≥30% decrease in sum longest distance relative to baseline	<30% decrease relative to baseline but <20% increase in sum longest distance relative to nadir	≥20% increase in sum longest distance relative to nadir*
Non-target lesions	None	Stable or improved	Stable or improved	Unequivocal progressive disease*
New lesion(s)†	None	None	None	Present*
Corticosteroids	None	Stable or decreased	Stable or decreased	Not applicable‡
Clinical status	Stable or improved	Stable or improved	Stable or improved	Worse*
Requirement for response	All	All	All	Any‡

*Progression occurs when this criterion is met. †A new lesion is one that not present on prior scans and is visible in minimum two projections. If a new lesion is equivocal, for example because of its small size, continued therapy can be considered, and follow-up assessment will clarify if the new lesion is new disease. If repeat scans confirm there is definitely a new lesion, progression should be declared using the date of the initial scan showing the new lesion. For immunotherapy-based approaches, new lesions alone to do not define progression. ‡Increase in corticosteroids alone will not be taken into account in determining progression in the absence of persistent clinical deterioration.

Table 2 CNS and non-CNS response assessment

CNS (RANO-BM)	Non-CNS (RECIST 1.1)	Response
Complete response, partial response, or stable disease	Complete response, partial response, or stable disease	Log as CNS and non-CNS complete response, partial response, or stable diseases
Complete response, partial response, or stable disease	Progressive disease	Log as CNS complete response, partial response, or stable disease; log as non-CNS progressive disease
Progressive disease	Complete response, partial response, or stable disease	Log as CNS progressive disease; log as non-CNS complete response, partial response, or stable disease
Progressive disease	Progressive disease	Log as both CNS and non-CNS progressive disease

Table 3 Bi-compartmental progression-free survival

CNS (RANO-BM)	Non-CNS (RECIST 1·1)	Bi-compartmental PFS	Note
Complete response, partial response, or stable disease	Progressive disease	Log as a progression-free survival event	Log as non-CNS progressive disease
Progressive disease	Complete response, partial response, or stable disease	Log as a progression-free survival event	Log as CNS progressive disease
Progressive disease	Progressive disease	Log as a progression-free survival event	Log as both CNS and non-CNS progressive disease
