# Supplementary material for: Phase II Clinical Study of Adebrelimab and Bevacizumab Combined With Cisplatin/Carboplatin in Patients With Triple-Negative Breast Cancer With Brain Metastases (ABC Study)
Source: J Clin Oncol. 2026 Mar 4;44(26):2543–53. doi: 10.1200/JCO-25-02021 (PMC13557479; doi:10.1200/JCO-25-02021)
Supplement: Supplementary file 2 [file jco-44-2543-s002.pdf]

STATISTICAL ANALYSIS PLAN APPROVAL PAGE

Protocol Title:

A Phase II Clinical Study of Adebrelimab and Bevacizumab Combined with Cisplatin/Carboplatin in Triple-Negative Breast Cancer Patients with Brain Metastases

SAP Version: 2.0

Date: 13 May 2024

1 Introduction 3

1.1 Study Design 3

1.2 Sample Size 3

2 Study Endpoints 4

2.1 Efficacy Endpoints 4

2.1.1 Objective response rate in the central nervous system (CNS-ORR) 4

2.1.2 Clinical benefit rate in the central nervous system (CNS-CBR) 4

2.1.3 Progression-free progress (PFS)* 4

2.1.4 Overall survival (OS) 4

2.1.5 Site of First Progression 4

2.2 Safety Endpoints 4

2.2.1 Adverse Events 4

2.2.2 Laboratory Test 5

2.2.3 Physical Examination and Vital Signs 5

2.2.4 Electrocardiogram and Echocardiography 5

2.3 Exploratory Endpoints 5

3 Statistical Analysis 5

3.1 Analysis Sets 5

3.2 Method of Statistics 6

3.2.1 Subject Disposition and Demographics 6

3.2.2 Efficacy Analysis 6

3.2.3 Safety analysis 6

3.2.4 Exploratory analysis 6

4 References 7

1 Introduction

This Statistical Analysis Plan (SAP) details the statistical methods to be used in the analysis of this study. This SAP does not include tables, listings, and figures (TLFs), which will be presented in a separate document. Any changes to the statistical methods will require an amendment to the SAP.

This SAP is based on the following documents: Study Protocol, Version 2.0 (13 May 2024).

1.1 Study Design

This is an open-label, prospective, single-arm, single-center phase II clinical trial. The study plans to enroll 35 patients diagnosed with triple-negative breast cancer (TNBC) and brain metastases. Key eligibility criteria included:

1. No prior use of bevacizumab or anti-PD-1/PD-L1 agents.
2. Prior platinum therapy is permitted only in platinum-sensitive disease (defined as no progression during within 4 platinum cycles and a treatment-free interval more than 3 months before progression).

Eligible patients will receive combination therapy every 21 days, administered sequentially on day 1 of each cycle in the following order:

- Adebrelimab (anti-PD-L1 antibody; 20 mg/kg IV infusion),
- Bevacizumab (7.5 mg/kg IV infusion),
- Cisplatin (75 mg/m²) or Carboplatin (AUC=5).

Treatment continues until disease progression, unacceptable toxicity, withdrawal of informed consent, or discontinuation of the study as determined by the investigators, whichever occurs first.
Follow-up:

1. Safety follow-up: until 28 days after the last dose. Follow-up should continue until the AE is resolved, the subject starts a new antitumor therapy, or the condition is deemed irreversibly stable by the researcher.

2. Efficacy follow-up (for subjects discontinuing without progression): until progression, new anti-tumor therapy, or death.

3. Overall survival (OS) follow-up: every 3 months until death, loss to follow-up, or study completion.
The study flowchart is detailed in the protocol. The study design diagram is shown below:

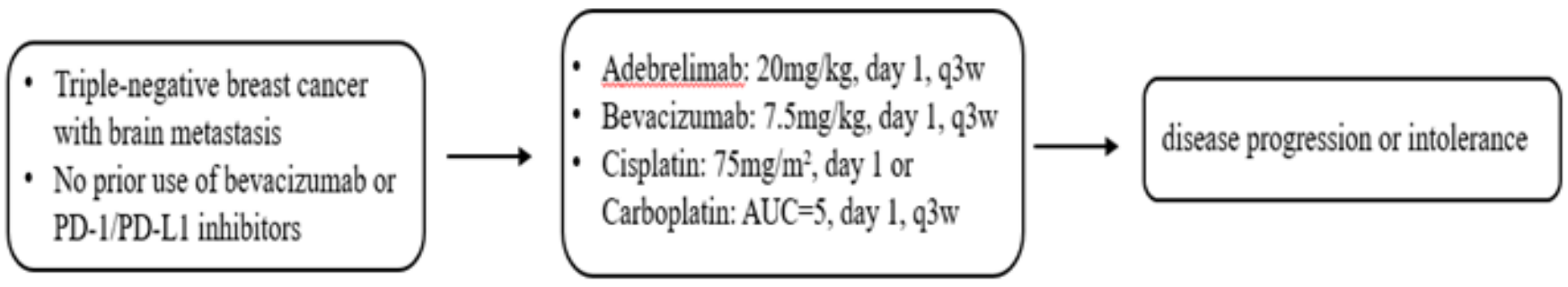

1.2 Sample Size

The sample size was calculated based on the primary endpoint of CNS-ORR using a Simon's optimal two-stage design (Simon, 1989)[1]. Since the limited data reported at the time of ABC study design, referring to

the historical data on platinum-based regimen in solid tumor BMs and iniparib-based regimen in TNBC BMs, the null hypothesis for CNS-ORR was set at 14%[2,3], and the alternative hypothesis for CNS-ORR was set at 34% (one-sided α=0.05, power=80%).

- Stage 1: enroll 13 subjects. If ≤2 responses are observed, the trial will be stopped for futility.
- Stage 2: enroll an additional 18 subjects (total n=31). If ≤7 total responses are observed, the null hypothesis is not rejected. If >7 responses are observed, the null hypothesis is rejected in favor of efficacy.

Accounting for a 10% dropout rate, 35 subjects will be enrolled.

2 Study Endpoints

2.1 Efficacy Endpoints

2.1.1 Objective response rate in the central nervous system (CNS-ORR)

The primary endpoint was objective response rate in the CNS (CNS-ORR), defined as the proportion of patients with the best intracranial response of complete response (CR) or partial response (PR) according to RANO-BM criteria by investigators.

2.1.2 Clinical benefit rate in the central nervous system (CNS-CBR)

The secondary endpoint CNS-CBR defined as the percentage of patients who experience an intracranial CR, PR or stable disease (SD) ≥ 24weeks per RANO-BM criteria.

2.1.3 Progression-free progress (PFS)*

The secondary endpoint PFS defined as the time from the first dose of treatment to death or disease progression.

*PFS including overall PFS (assessed by RANO-BM criteria and RECIST 1.1 criteria) and CNS-PFS (assessed by RANO-BM criteria).

2.1.4 Overall survival (OS)

The secondary endpoint OS defined as the time from the first dose of treatment to death for any cause.

2.1.5 Site of First Progression

The secondary endpoint Site of First Progression defined as the first lesion to progress.

2.2 Safety Endpoints

The safety of the study drug will be evaluated through the recording of adverse events (including serious adverse events), laboratory tests, vital signs, physical examinations, ECOG scores, echocardiography, and electrocardiograms during the trial period.

2.2.1 Adverse Events

The incidence and grade of adverse events (AE) and severe adverse events (SAE); AE is assessed according to the NCI-CTCAE 5.0.

AE refers to an unfavorable medical occurrence that appears after a subject in a clinical trial, but it does not necessarily have a causal relationship with the treatment. AE can be any unfavorable and unintended symptoms, signs, abnormal laboratory findings, or diseases.

SAE refers to a medical occurrence during a clinical trial that requires hospitalization or prolongs hospitalization, results in disability, affects the ability to work, is life-threatening or results in death, or causes congenital anomalies.

The collection of AE information begins when the subject signs the informed consent form and continues until the end of the safety follow-up period (28 days after the last dose of medication). Follow-up should continue until the AE is resolved, the subject starts a new antitumor therapy, or the condition is deemed irreversibly stable by the researcher.

2.2.2 Laboratory Test

Standard safety laboratory parameters will be summarized. Values outside reference ranges or clinically significant shifts from baseline will be graded per NCI-CTCAE 5.0.

2.2.3 Physical Examination and Vital Signs

Physical examination: includes general condition, skin and mucous membranes, lymph nodes, head and neck, chest, abdomen, musculoskeletal system, neurological reflexes, respiratory system, cardiovascular system, genitourinary system, and mental state. Weight is measured at each exam; height is measured once at screening.

Vital signs: temperature, blood pressure, respiratory rate, pulse (per protocol schedule).

2.2.4 Electrocardiogram and Echocardiography

The 12-lead electrocardiogram and Echocardiography will be performed by qualified personnel at protocol-specified timepoints.

2.3 Exploratory Endpoints

Analysis of predictive/prognostic biomarkers (tissue/blood) and their association with treatment response or disease status.

3 Statistical Analysis

The analysis plan will be finalized before database lock. Statistical reports will follow data analysis.

3.1 Analysis Sets

The analysis population for this study includes the Full Analysis Set (FAS), the Per Protocol Set (PPS), and the Safety Set (SS).

- FAS: all subjects who received the study drug at least once will be included in the FAS. The full analysis set is the primary analysis set used for the efficacy analysis of this study.
- PPS: the PPS is a subset of the FAS, defined as subjects who have at least one medication record and have not experienced major protocol violations deemed to affect the efficacy analysis.
- SS: includes all enrolled subjects who have at least one medication record.

3.2 Method of Statistics

Unless otherwise specified, the data in this study will be described and summarized following the general principles below.

- Categorical data: frequency (n), percentage (%), 95% confidence intervals (CI) via Clopper-Pearson method[4].
- Continuous data: median, minimum, and maximum values.
- Time-event data: Kaplan-Meier estimates (median, 95% CI via Brookmeyer-Crowley method[5]).

3.2.1 Subject Disposition and Demographics

The descriptive statistics of subject disposition and demographics and baseline data will be summarized based on FAS.

- Screening status (screened, screen failures, treated).
- Treatment status (ongoing, discontinued, reason).
- Study completion/withdrawal (status, reason).

3.2.2 Efficacy Analysis

- CNS-ORR: Estimated proportion with 95% CI for FAS and PPS.
- CNS-CBR: Proportion with 95% CI.
- PFS/OS: Kaplan-Meier curves, median with 95% CI.
- Site of First Progression: Frequency by location.

3.2.3 Safety analysis

Summarized for SS:

- AE/SAE incidence, severity, and relationship to treatment.
- Laboratory shifts, vital sign changes, electrocardiograms， echocardiography.

3.2.4 Exploratory analysis

The exploratory analysis will be based on the FAS and PPS.

4 References

1. Simon R: Optimal two-stage designs for phase II clinical trials. Control Clin Trials 10:1-10, 1989
2. Viñolas N, Graus F, Mellado B, et al: Phase II trial of cisplatinum and etoposide in brain metastases of solid tumors. J Neurooncol 35:145-8, 1997
3. Anders C, Deal AM, Abramson V, et al: TBCRC 018: phase II study of iniparib in combination with irinotecan to treat progressive triple negative breast cancer brain metastases. Breast Cancer Res Treat 146:557-66, 2014
4. Clopper CJ, Pearson ES: The Use of Confidence or Fiducial Limits Illustrated in the Case of the Binomial. Biometrika 26:404-413, 1934
5. Brookmeyer R, Crowley J: A Confidence Interval for the Median Survival Time. Biometrics 38:29-41, 1982
